# Sequential two-step, one-pot microwave-assisted Urech synthesis of 5-monosubstituted hydantoins from L-amino acids in water

**DOI:** 10.3762/bjoc.21.46

**Published:** 2025-03-14

**Authors:** Wei-Jin Chang, Sook Yee Liew, Thomas Kurz, Siow-Ping Tan

**Affiliations:** 1 Department of Physical Science, Faculty of Applied Sciences, Tunku Abdul Rahman University of Management and Technology, 53300 Kuala Lumpur, Malaysia; 2 Centre for Foundation Studies, University of Malaya, 50603 Kuala Lumpur, Malaysia; 3 Institute of Pharmaceutical and Medicinal Chemistry, Heinrich Heine University Düsseldorf, 40225 Düsseldorf, Germany

**Keywords:** amino acids, hydantoin, microwave-assisted, one-pot reaction, Urech synthesis

## Abstract

The hydantoin scaffold is renowned for its wide-ranging biological activities, including antibacterial, antiviral, anticancer, anti-inflammatory, and anticonvulsant effects. In this study, we present an innovative, sustainable approach to synthesizing hydantoins (**H2a**–**j**) directly from amino acids. This method employs a column chromatography-free, two-step, one-pot microwave-assisted synthesis that delivers hydantoins in yields ranging from 34% to 89%. The protocol demonstrates exceptional functional group tolerance, accommodating phenyl, aliphatic, phenol, alcohol, heterocyclic, and sulfide groups. This scalable, rapid, and eco-friendly strategy offers a promising avenue for the efficient synthesis of hydantoins, aligning with green chemistry principles and expanding the accessibility of these bioactive compounds for pharmaceutical applications.

## Introduction

The hydantoin moiety is a scaffold found in many biologically active compounds exhibiting a diverse range of properties, including antibacterial [1], antiviral [2], anticancer [3], anti-inflammatory [4], and anticonvulsant [5]. Hydantoins were traditionally accessed from amino acids by conversion to urea derivatives followed by cyclization (Urech reaction) or from carbonyl compounds through the cyanohydrin intermediate (Bucherer–Bergs) [6]. Furthermore, several methods for hydantoin synthesis have been reported. The modified Bucherer–Bergs reaction using nitriles or methyleneaziridines with organometallic reagents gave moderate yields of 40–77% and 48–75%, respectively [7–8]. Amino acids remain key building blocks, with approaches involving methylated amino acids and carbamates (65–75%) [9] or cyanobenziodoxolone (CBX) (54–88%) [10]. Reactions using 1,2-diaza-1,3-dienes, amines, and isocyanates (47–76%) [11], as well as α-amino amides with triphosgene or carbonyldiimidazole (CDI) (41–80%) [12], have also been explored. Other strategies include copper(I)-catalyzed reactions (48–71%) [13] and the four-component Ugi reaction [14] yields 48–71% and 27–67%, respectively. In addition, pyroglutamates with isocyanates, which provide exceptionally high yields (97–99%) [15], and dipeptides have been utilized in triflate-activated Mumm’s rearrangement (63–86%) [16]. As many of these protocols employ toxic, highly reactive and moisture-sensitive reagents, our work will exploit microwave technology and the environmentally benign nature of amino acids to construct the hydantoin scaffold. Microwave irradiation technology has proven significant advantages over conventional heating, such as enhanced reaction rates and yields [17]. Microwave irradiation is also aligned with our group’s sustainability goals of adhering to the principles of green chemistry [18].

Following our previous works in the synthesis and evaluation of biologically active hydantoins [19–20], we aim towards creating a more efficient procedure to access hydantoins. We are interested in the synthesis of hydantoins from amino acids and potassium cyanate in water due to its simplicity and environmental friendliness, which also precludes the use of water-sensitive and highly toxic reagents in the procedures mentioned above. Amino acids can be procured from natural sources by enzymatic synthesis [21], as well as synthetically accessed from aldehyde building blocks via the Strecker synthesis [22], making them attractive starting materials for hydantoin synthesis.

## Results and Discussion

**Scheme 1:**
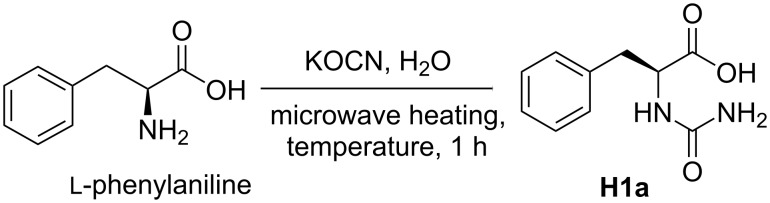
*N*-Carbamylation of ʟ-phenylaniline using KOCN in water.

As the first step of Urech hydantoin synthesis involved the *N*-carbamylation of amino acids, we carried out the microwave-assisted synthesis of urea derivatives in water, utilizing ʟ-phenylaniline as the representative amino acid. The optimal conditions for the *N*-carbamylation of ʟ-phenylaniline were first verified using different equivalents of KOCN at different temperatures in water under microwave heating conditions (Scheme 1, Table 1), and 5.0 equiv of KOCN and microwave irradiation at 80 °C produced the best yield for **H1a** (Table 1, entry 2).

**Table 1:** Optimization of *N*-carbamylation of **H1a**.

Entry	KOCN	Temperature^a^	Yield of **H1a**^b^

1	4.0 equiv	80 °C	70%
2	5.0 equiv	80 °C	89%
3	6.0 equiv	80 °C	61%
4	5.0 equiv	60 °C	64%
5	5.0 equiv	100 °C	60%

^a^Reaction mixture was heated in an Anton–Parr Monowave 400 microwave reactor with an infra-red sensor. ^b^Isolated yield. Reaction was carried out at 5 mmol scale.

**Scheme 2:**
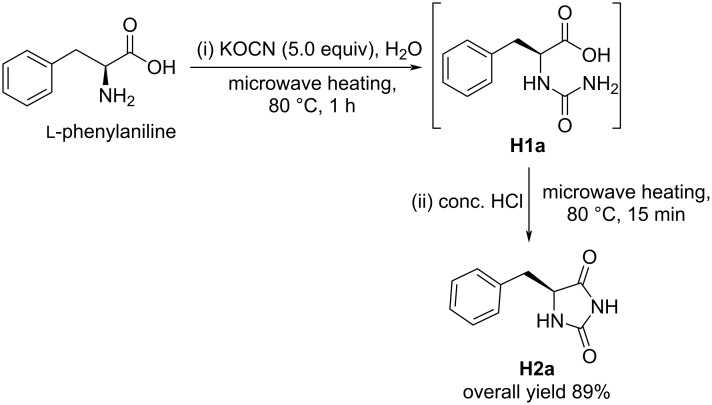
One-pot microwave-assisted synthesis of hydantoins from amino acids.

Inspired by the simplicity of the procedure, a second step was attempted as part of a one-pot synthesis of hydantoins by the addition of concentrated hydrochloric acid followed by microwave irradiation of the reaction mixture at 80 °C for 15 min (Scheme 2). Gratifyingly, the acid-induced intra-cyclization of the urea derivative **H1a** proceeded smoothly to give hydantoin **H2a** with an overall yield of 89%, which indicated complete conversion of the intermediate to the product.

**Figure 1:**
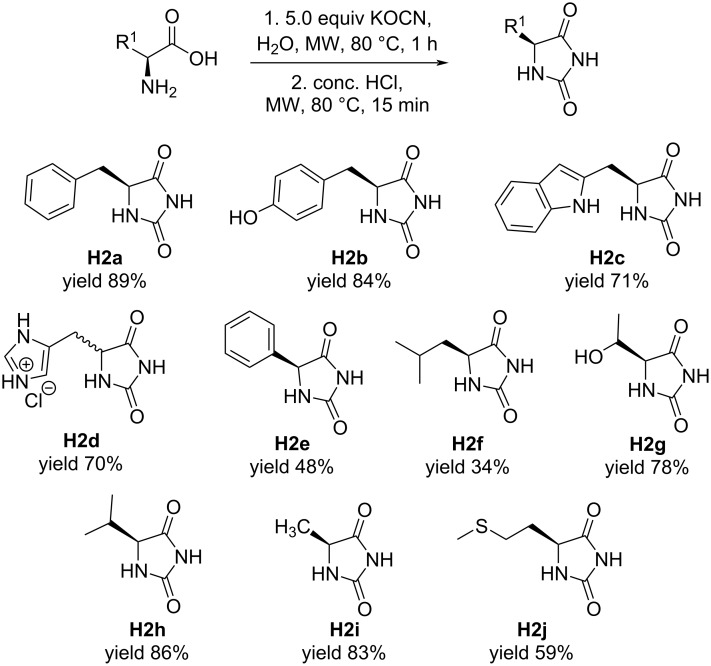
Hydantoins (**H2a–j**) synthesized from the one-pot procedure. The hydantoins were characterized using ^1^H and ^13^C NMR and HRMS.

Encouraged by the result of the one-pot procedure, we tested the method with amino acids carrying different functional groups on the side chains (Figure 1). Phenol (**H2b**), isopropyl (**H2h**) and methyl groups (**H2i**) were well-tolerated in the reaction, yielding excellent and consistent conversions of the amino acids to the corresponding hydantoins (83–86%). However, the other aliphatic group – isobutyl – was poorly tolerated, giving a low yield (34%) of **H2f**. This could be attributed to the methyl group in the isobutyl side chain projected into the plane of the hydantoin ring, where the end methyl groups are conformationally positioned to hinder the reaction center. In the case where a phenyl group is directly connected to the reaction center – as opposed to a benzylic attachment – a near two-fold decrease in yield was observed (**H2a** vs **H2e**), inferring that the reaction is strongly affected by steric effects. A high yield (78%) was also observed for the hydroxy-bearing hydantoin from threonine (**H2g**), suggesting that the reaction works smoothly in the presence of an electron-donating protic group close to the reaction center. Good yields (70–71%) of hydantoins containing heterocyclic groups, such as indole (**H2c**) and imidazole (**H2d**) were also achieved. This demonstrates potential application of this protocol in the synthesis of pharmacologically active hydantoin compounds. The conversion of the thioether-containing methionine to hydantoin (**H2j**) was obtained with a moderate yield of 59%. Lastly, no hydantoin product was observed by ^1^H NMR when cysteine was subject to the one-pot procedure, possibly due to disulfide formation of the highly nucleophilic thiol group of the cysteine amino acid. The hydantoins were found to be optically active (except for **H2d**), suggesting that enantiomeric information was preserved during the reaction (see Supporting Information File 1). Hydantoin yields (34–89%) are generally comparable to previously reported methods [7–16], but reaction times are significantly shorter. In addition, TLC analysis confirmed complete consumption of starting material, which was further confirmed by NMR analysis showing no detectable impurities or unreacted starting material. Variations in yields (34–89%) may be due to differences in substrate reactivity, minor side reactions, or minor losses during microwave irradiation. The remaining part may be caused by factors such as slight volatilization or incomplete conversion under specific use conditions.

We found the synthetic procedure facile as the less polar hydantoin products (**H2a–c**, **H2e**,**f**) often precipitated out of the reaction mixture and were sufficiently pure (>95% by HPLC) after the simple washing steps, and the more polar hydantoin products could be extracted out of the reaction mixture with ethyl acetate and did not require further chromatographic purification. The “one-pot” aspect of the protocol under microwave irradiation conditions, which enables rapid conversion of the starting materials to the products in water – a green solvent – in less than two hours makes this protocol an expedient and environmentally benign synthesis of hydantoins from amino acids.

The scalability of this process is promising, driven by its simplicity, moderate to high yields, and the use of environmentally friendly reagents. Notably, microwave-assisted one-pot reactions eliminate the need for column chromatography, a common step in traditional purification methods but limits scalability. Additionally, the reaction proceeds efficiently without the need for toxic or moisture-sensitive reagents, further making it suitable for large-scale applications. The shorter reaction time (less than 2 hours) also contributes to improve energy efficiency when compared to traditional methods. These factors demonstrate that the process can be effectively scaled up, particularly in industries that prioritize sustainability and operational efficiency.

In terms of green chemistry principles, this approach already meets several key goals, such as reducing the use of hazardous reagents and solvents, increasing reaction efficiency, and minimizing waste. Future studies can quantitatively evaluate these aspects using established metrics such as E-factor, atom economy, and reaction mass efficiency. These indicators will provide a more comprehensive evaluation of the environmental sustainability of the reaction and further demonstrate its potential for large-scale, environmentally responsible production.

## Conclusion

In conclusion, we report a facile, environmentally friendly, chromatography-free hydantoin synthesis method using a sequential two-step, one-pot microwave-assisted method. This strategy utilizes amino acids as precursors and achieves yields of 34–89%, with a broad functional group tolerance for phenyl, aliphatic, phenol, alcohol, heterocyclic, and sulfide groups. By eliminating highly reactive and moisture-sensitive reagents, our method improves practicality and safety, while microwave irradiation significantly speeds up reaction times, making it a valuable advancement in green and sustainable chemistry.

## Experimental

### General procedure for the synthesis of (*S*)-5-benzylimidazolidine-2,4-dione (**H2a**)

A 30 mL microwave reactor vial was charged with ʟ-phenylalanine (5 mmol), distilled water (7 mL), and potassium cyanate (25 mmol) and irradiated in an Anton–Paar Monowave 400 microwave reactor at 80 °C for 1 hour. Upon completion of the *N*-carbamylation reaction, as confirmed by TLC analysis, the reaction mixture was treated with concentrated hydrochloric acid (7 mL) and microwave irradiated at 80 °C for 15 min. The precipitates in the reaction mixture were filtered, washed with 1 M HCl solution (2 × 7 mL), distilled water (2 × 10 mL), and dried to afford **H2a** as a white solid. Yield 89%. [α]_D_^20^ −100.9 (*c* 11, CH_3_CN); ^1^H NMR (400 MHz, DMSO-*d*_6_) δ 10.44 (s, 1H), 7.93 (s, 1H), 7.17–7.30 (m, *J* = 8 Hz, 5H), 4.33 (td, *J* = 5, 1 Hz, 1H), 2.92 (m, *J* = 5 Hz, 2H); ^13^C NMR (100 MHz, DMSO-*d*_6_) δ 175.2, 157.2, 135.6, 129.8, 128.1, 126.7, 58.4, 36.4; LC–MS (ESI) *m*/*z*: 191 [M + H]^+^, 163, 120; HRMS (ESI) *m*/*z*: [M + H]^+^ calcd for C_10_H_11_N_2_O_2_ 191.0815; found, 191.0815. The procedure was repeated by substituting ʟ-phenylalanine with the corresponding amino acid to obtain **H2b**,**c** and **H2e**,**f**.

### General procedure for the synthesis of (*S*)-5-((2,5-dioxoimidazolidin-4-yl)methyl)-1*H*-imidazol-3-ium chloride (**H2d**)

A 30 mL microwave reactor vial was charged with ʟ-histidine (5 mmol), distilled water (7 mL), and potassium cyanate (25 mmol) and irradiated in an Anton–Paar Monowave 400 microwave reactor at 80 °C for 1 hour. Upon completion of the *N*-carbamylation reaction, as confirmed by TLC analysis, the reaction mixture was treated with concentrated hydrochloric acid (7 mL) and microwave irradiated at 80 °C for 15 min. The reaction mixture was neutralized to pH 7 using saturated sodium bicarbonate solution and extracted with ethyl acetate multiple times. The organic extracts were combined, dried with anhydrous sodium sulfate, and concentrated under reduced pressure to afford **H2d** as a white solid. Yield 70%. ^1^H NMR (400 MHz, DMSO-*d*_6_) δ 10.59 (s, 1H), 7.88 (s, 1H), 7.74 (s, 1H), 6.88 (s, 1H), 5.52 (s, 2H), 4.25 (t, *J* = 6 Hz, 1H), 2.95 (dd, *J* = 15, 4 Hz, 1H), 2.81 (dd, *J* = 15, 6 Hz, 1H); ^13^C NMR (100 MHz, DMSO-*d*_6_) δ 175.5, 157.4, 134.6, 131.6, 117.0, 57.6, 28.8; LC–MS (ESI) *m*/*z*: 181 [M + H]^+^, 136. The procedure was repeated by substituting ʟ-histidine with the corresponding amino acid to obtain **H2g–j**.

## Supporting Information

File 1 NMR and MS spectra of synthesized compounds.

## Data Availability

All data that supports the findings of this study is available in the published article and/or the supporting information of this article.
