# Supplementary material for: Sequential two-step, one-pot microwave-assisted Urech synthesis of 5-monosubstituted hydantoins from L-amino acids in water
Source: Beilstein J Org Chem. 2025 Mar 14;21:596–600. doi: 10.3762/bjoc.21.46 (PMC11912639; doi:10.3762/bjoc.21.46)
Supplement: File 1 — NMR and MS spectra of synthesized compounds. [file Beilstein_J_Org_Chem-21-596-s001.pdf]

BEILSTEIN JOURNAL OF ORGANIC CHEMISTRY

Supporting Information

for

Sequential two-step, one-pot microwave-assisted Urech synthesis of 5-monosubstituted hydantoins from L-amino acids in water

Wei-Jin Chang, Sook Yee Liew, Thomas Kurz and Siow-Ping Tan

Beilstein J. Org. Chem. **2025,** *21,* 596–600. doi:10.3762/bjoc.21.46

NMR and MS spectra of synthesized compounds

License and Terms: This is a supporting information file under the terms of the Creative Commons Attribution License (https://creativecommons.org/licenses/by/4.0). Please note that the reuse, redistribution and reproduction in particular requires that the author(s) and source are credited and that individual graphics may be subject to special legal provisions.
The license is subject to the *Beilstein Journal of Organic Chemistry* terms and conditions: (https://www.beilstein-journals.org/bjoc/terms)

Supporting Information

Table of contents

1. General information S1

2. Synthesis and characterization of products S1

2.1 Characterization data S2

3. NMR and HRMS spectra S3

4. Chromatograms of compounds S22

5. References S27

1. General information

All chemicals were acquired from commercial sources (Merck or Thermo Fischer) at reagent grade and were used without further purification. The syntheses were carried out in an Anton-Parr Monowave 400 microwave reactor with the reaction temperature controlled using the on-broad infra-red sensor. The NMR spectra were acquired in a Bruker DMX 400 NMR instrument at a resolution of 400 MHz and 100 MHz, for ^{1}H and ^{13}C NMR respectively, using the residual solvent peak of DMSO-d_6 at 2.50 ppm (^{1}H NMR) and 39.5 ppm (^{13}C NMR) as reference. All chemical shifts (δ) were recorded in ppm unit and coupling frequency (*J*) in Hertz (Hz). Shorthand notations were used for the NMR characterization: s (singlet); d (doublet); q (quartet); dd (doublet of doublets); td (triplet of doublets); m (multiplet). The high resolution *m*/*z* values were measured in Dalton units (Da) using an Agilent 1290 Infinity LC system coupled to an Agilent 6520 Accurate-Mass Q-TOF mass spectrometer with the ESI source in positive mode. Shorthand notation: calcd (calculated). HPLC analyses were performed using a mobile phase of acetonitrile/ultrapure water (9:1) in an Agilent 1260 with a Zorbax C_{18} column (5 µm) and detected by diode array at 210 nm and 254 nm. All hydantoin compounds were reported to be >95% pure.

2. Synthesis and characterization of products

General procedure for the synthesis of (*S*)-5-benzylimidazolidine-2,4-dione (**H2a**):

A 30 mL microwave reactor vial was charged with L-phenylalanine (5 mmol), distilled water (7 mL), and potassium cyanate (25 mmol) and irradiated in an Anton–Paar Monowave 400 microwave reactor at 80 °C for 1 hour. Upon completion of the *N*-carbamylation reaction, as confirmed by TLC analysis, the reaction mixture was treated with concentrated hydrochloric acid (7 mL) and microwave irradiated at 80 °C for 15 min. The precipitates in the reaction mixture were filtered, washed with 1 M HCl solution (2 × 7 mL), distilled water (2 × 10 mL), and dried to afford **H2a** as a white solid. Yield 89%. $[\alpha]_D^{20}$ -100.9 (*c* 11, CH_3CN). ^{1}H NMR (400 MHz, DMSO-d_6) δ 10.44 (s, 1H), 7.93 (s, 1H), 7.17 – 7.30 (m, 5H), 4.33 (td, *J* = 5, 1 Hz, 1H), 2.92 (m, 2H). ^{13}C NMR (100 MHz, DMSO-d_6) δ 175.2, 157.2, 135.6, 129.8, 128.1, 126.7, 58.4, 36.4. LC-MS (ESI) m/z: 191 $[M+H]^+$, 163, 120. HRMS (ESI) m/z: $[M+H]^+$ Calcd for $C_{10}H_{11}N_2O_2$ 191.0815; Found: 191.0815. The procedure was repeated by substituting L-phenylalanine with the corresponding amino acid to obtain **H2b–c** and **H2e–f**.

General procedure for the synthesis of (*S*)-5-((2,5-dioxoimidazolidin-4-yl)methyl)-1*H*-imidazol-3-ium chloride (**H2d**):

A 30 mL microwave reactor vial was charged with L-histidine (5 mmol), distilled water (7 mL), and potassium cyanate (25 mmol) and irradiated in an Anton-Paar Monowave 400 microwave reactor at 80 °C for 1 hour. Upon completion of the *N*-carbamylation reaction, as confirmed by TLC analysis, the

reaction mixture was treated with concentrated hydrochloric acid (7 mL) and microwave irradiated at 80 °C for 15 min. The reaction mixture was neutralized to pH 7 using saturated sodium bicarbonate solution and extracted with ethyl acetate multiple times. The organic extracts were combined, dried with anhydrous sodium sulfate, and concentrated under reduced pressure to afford **H2d** as a white solid. Yield 70%. ^{1}H NMR (400 MHz, DMSO-d_6) δ 10.59 (s, 1H), 7.88 (s, 1H), 7.74 (s, 1H), 6.88 (s, 1H), 5.52 (s, 2H), 4.25 (t, *J* = 6 Hz, 1H), 2.95 (dd, *J* = 15, 4 Hz, 1H), 2.81 (dd, *J* = 15, 6 Hz, 1H). ^{13}C NMR (100 MHz, DMSO-d_6) δ 175.5, 157.4, 134.6, 131.6, 117.0, 57.6, 28.8. LC-MS (ESI) m/z: 181 $[M+H]^+$, 136. The procedure was repeated by substituting L-histidine with the corresponding amino acid to obtain **H2g–j**.

2.1 Characterization data

Carbamoyl-L-phenylalanine (**H1a**):

White solid. Yield 89%. $[\alpha]_D^{20}$ -33.4 (*c* 10, CH_3CN). ^{1}H NMR (400 MHz, DMSO-d_6) δ 7.28 (t, *J* = 7 Hz, 2H), 7.20 (m, 3H), 6.18 (d, *J* = 8 Hz, 1H), 5.62 (s, 2H), 4.33 (dd, *J* = 13, 8 Hz, 1H), 2.99 (dd, *J* = 13, 5 Hz, 1H), 2.84 (dd, *J* = 13, 8 Hz, 1H). ^{13}C NMR (100 MHz, DMSO-d_6) δ 174.1, 158.4, 137.6, 129.4, 128.3, 126.6, 53.9, 37.7. LC-MS (ESI) m/z: 209 $[M+H]^+$, 166, 120. Data in accordance with literature [1].[20]

(*S*)-5-(4-Hydroxybenzyl)imidazolidine-2,4-dione (**H2b**):

White solid. Yield 84%. $[\alpha]_D^{20}$ -90.5 (*c* 10, acetone). ^{1}H NMR (400 MHz, DMSO-d_6) δ 10.39 (1H, s), 9.27 (s, 1H), 7.87 (s, 1H), 6.96 (d, *J* = 8 Hz, 2H), 6.64 (d, *J* = 8 Hz, 2H), 4.23 (q, *J* = 5 Hz, 1H), 2.80 (d, *J* = 5 Hz, 2H). ^{13}C NMR (100 MHz, DMSO-d_6) δ 175.4, 157.3, 156.1, 130.8, 125.5, 114.9, 58.7, 35.6. LC-MS (ESI) m/z: 207 $[M+H]^+$, 179, 136, 107. HRMS (ESI) m/z: $[M+H]^+$ Calcd for $C_{10}H_{11}N_2O_3$ 207.0764; Found: 207.0759.

(*S*)-5-((1*H*-Indol-2-yl)methyl)imidazolidine-2,4-dione (**H2c**):

White solid. Yield 71%. $[\alpha]_D^{20}$ -97.1 (*c* 10, CH_3CN). ^{1}H NMR (400 MHz, DMSO-d_6) δ 10.90 (s, 1H), 10.37 (s, 1H), 7.91 (s, 1H), 7.55 (d, *J* = 8 Hz, 1H), 7.32 (d, *J* = 8 Hz, 1H), 7.12 (d, *J* = 2 Hz, 1H), 7.05 (td, *J* = 7, 1 Hz, 1H), 6.97 (td, *J* = 7, 1 Hz, 1H), 4.30 (dd, *J* = 4, 1 Hz, 1H), 3.06 (d, *J* = 4 Hz, 2H). ^{13}C NMR (100 MHz, DMSO-d_6) δ 175.8, 157.5, 135.9, 127.5, 124.2, 120.9, 118.7, 118.4, 111.3, 108.0, 58.4, 26.5. LC-MS (ESI) m/z: 230 $[M+H]^+$, 130. HRMS (ESI) m/z: $[M+H]^+$ Calcd for $C_{12}H_{12}N_3O_2$ 230.0924; Found: 230.0926.

(*S*)-5-Phenylimidazolidine-2,4-dione (**H2e**):

White solid. Yield 48%. $[\alpha]_D^{20}$ -136.0 (*c* 10, CH_3CN). ^{1}H NMR (400 MHz, DMSO-d_6) δ 10.81 (s, 1H), 8.41 (s, 1H), 7.32 – 7.43 (m, 5H), 5.16 (s, 1H). ^{13}C NMR (100 MHz, DMSO-d_6) δ 174.3, 157.6, 136.1, 128.7, 128.3, 126.8, 61.3. LC-MS (ESI) m/z: 177 $[M+H]^+$, 149, 106. Data in accordance with literature [2].[21]

(*S*)-5-Isobutylimidazolidine-2,4-dione (**H2f**):

White solid. Yield 34%. $[\alpha]_D^{20}$ -78.0 (*c* 10, CH_3CN). ^{1}H NMR (400 MHz, DMSO-d_6) δ 10.59 (s, 1H), 8.01 (s, 1H), 3.99 (m, 1H), 1.75 (m, 1H), 1.34 – 1.51 (m, 2H), 0.87 (t, *J* = 6 Hz, 6H). ^{13}C NMR (100 MHz, DMSO-d_6) δ 176.6, 157.5, 56.2, 40.8, 24.2, 23.2, 21.5. LC-MS (ESI) m/z: 157 $[M+H]^+$, 129. Data in accordance with literature [2].[21]

(*S*)-5-((*R*)-1-Hydroxyethyl)imidazolidine-2,4-dione (**H2g**):

White solid. Yield 78%. $[\alpha]_D^{20}$ -103.4 (*c* 10, CH_3CN). ^{1}H NMR (400 MHz, DMSO-d_6) δ 7.89 (s, 1H), 5.48 (s, 1H), 4.93 (d, *J* = 6 Hz, 1H), 3.85 – 3.92 (m, 1H), 3.82 (dd, *J* = 3, 1 Hz, 1H), 1.12 (d, *J* = 6 Hz, 3H). ^{13}C NMR (100 MHz, DMSO-d_6) δ 174.8, 158.2, 65.2, 63.9, 20.3. LC-MS (ESI) m/z: 145 $[M+H]^+$, 101. Data in accordance with literature [3].[22]

(*S*)-5-Isopropylimidazolidine-2,4-dione (**H2h**):

White solid. Yield 86%. $[\alpha]_D^{20}$ -85.9 (*c* 10, CH_3CN). ^{1}H NMR (400 MHz, DMSO-d_6) δ 10.56 (s, 1H), 7.89 (s, 1H), 3.89 (dd, *J* = 4, 1 Hz, 1H), 1.94 – 2.02 (m, 1H), 0.93 (d, *J* = 7 Hz, 3H), 0.79 (d, *J* = 7 Hz, 3H). ^{13}C NMR (100 MHz, DMSO-d_6) δ 175.5, 157.9, 62.8, 29.6, 18.6, 15.9. LC-MS (ESI) m/z: 143 $[M+H]^+$, 115. HRMS (ESI) m/z: $[M+H]^+$ Calcd for $C_6H_{11}N_2O_2$ 143.0815; Found: 143.0814.

(*S*)-5-Methylimidazolidine-2,4-dione (**H2i**):

White solid. Yield 83%. $[\alpha]_D^{20}$ -23.2 (*c* 10, CH_3CN). ^{1}H NMR (400 MHz, DMSO-d_6) δ 10.58 (s, 1H), 7.88 (s, 1H), 4.02 (q, *J* = 7 Hz, 1H), 1.21 (d, *J* = 7 Hz, 3H). ^{13}C NMR (100 MHz, DMSO-d_6) δ 176.9, 157.2, 53.4, 17.3. LC-MS (ESI) m/z: 115 $[M+H]^+$. Data in accordance with literature [2].[21]

(*S*)-5-(2-(Methylthio)ethyl)imidazolidine-2,4-dione (**H2j**):

White solid. Yield 59%. $[\alpha]_D^{20}$ -49.9 (*c* 10, CH_3CN). ^{1}H NMR (400 MHz, DMSO-d_6) δ 10.66 (s, 1H), 8.02 (s, 1H), 4.10 (qd, *J* = 4, 1 Hz, 1H), 2.53 (m, 2H), 2.05 (s, 3H), 1.87 – 1.97 (m, 1H), 1.71 – 1.80 (m, 1H). ^{13}C NMR (100 MHz, DMSO-d_6) δ 175.9, 157.4, 56.6, 31.0, 28.8, 14.5. LC-MS (ESI) m/z: 175 $[M+H]^+$, 147, 127. Data in accordance with literature [2].[21]

3. NMR and HRMS Spectra

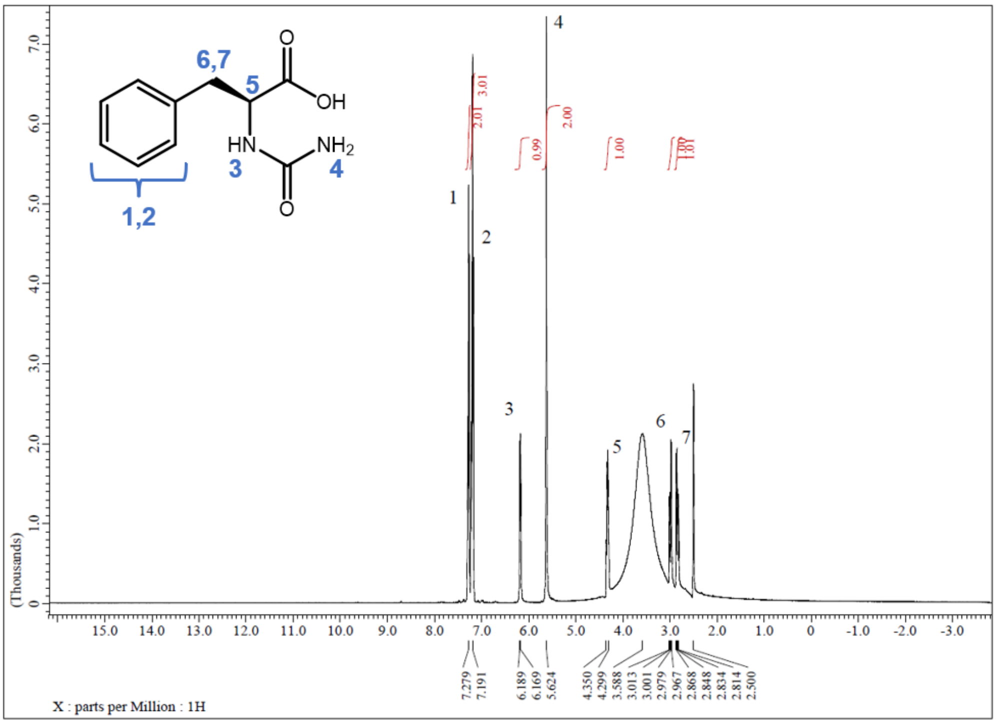

^{1}H NMR spectrum of carbamoyl-L-phenylalanine (**H1a**)

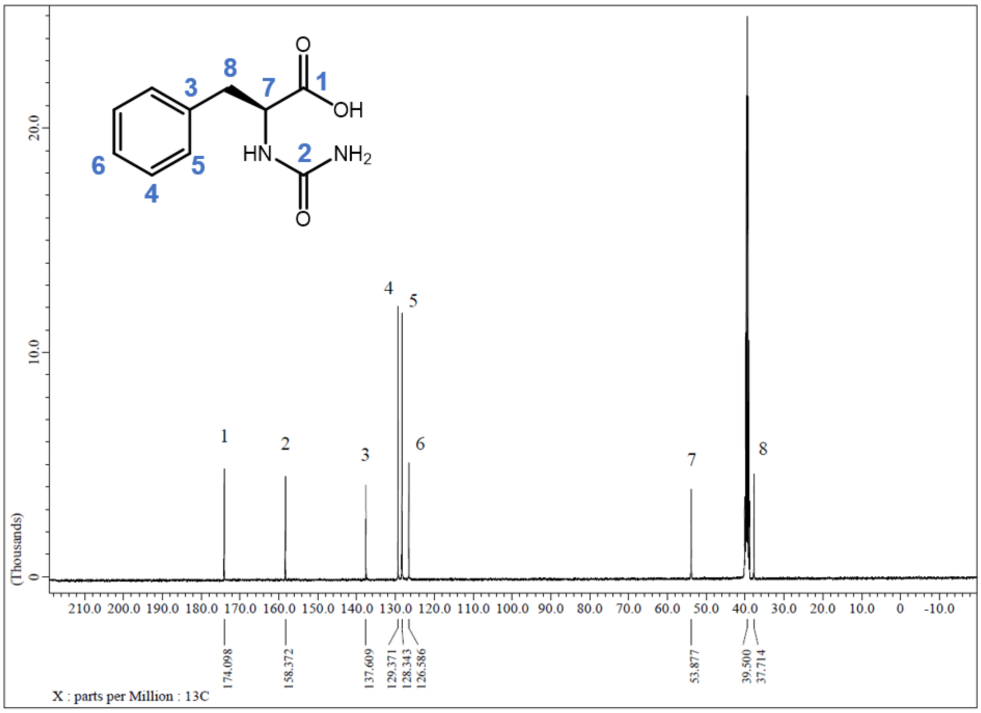

^{13}C NMR spectrum of carbamoyl-L-phenylalanine (**H1a**)

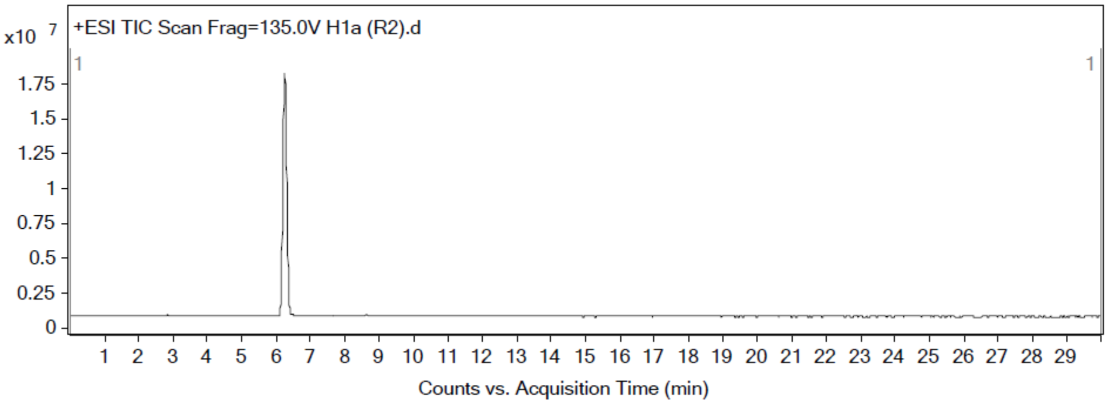

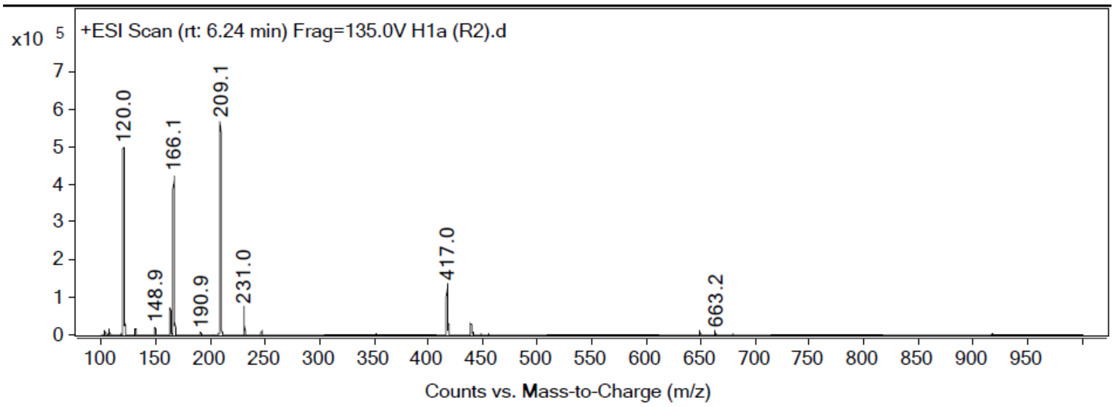

Mass spectrum of carbamoyl-L-phenylalanine (**H1a**)

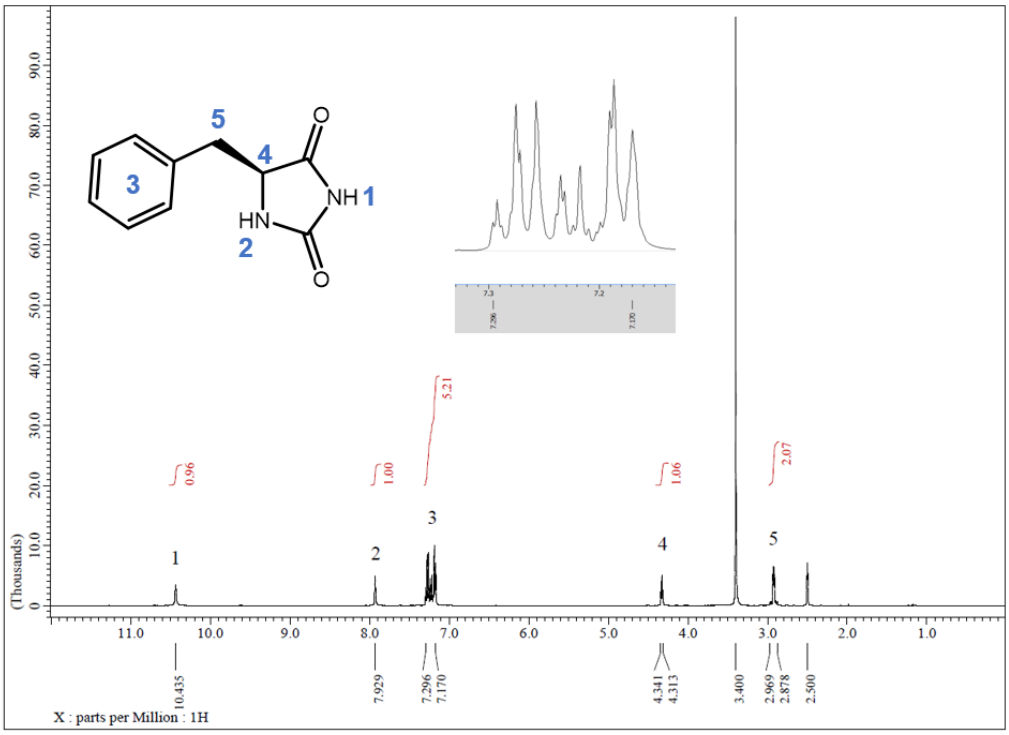

^{1}H NMR spectrum of (*S*)-5-benzylimidazolidine-2,4-dione (**H2a**)

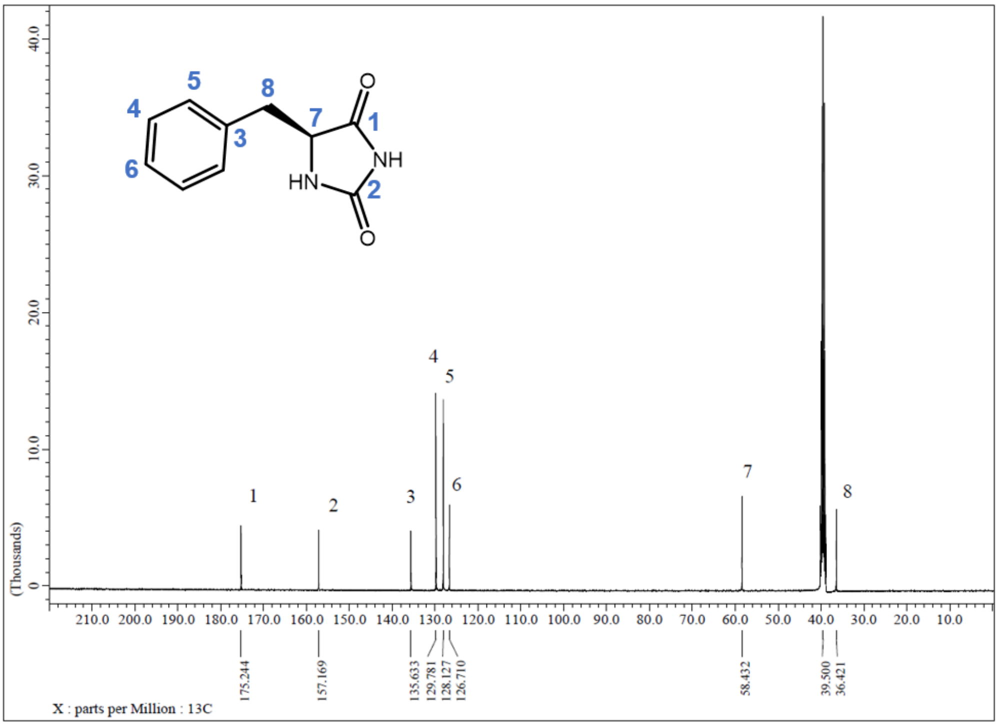

^{13}C NMR spectrum of (*S*)-5-benzylimidazolidine-2,4-dione (**H2a**)

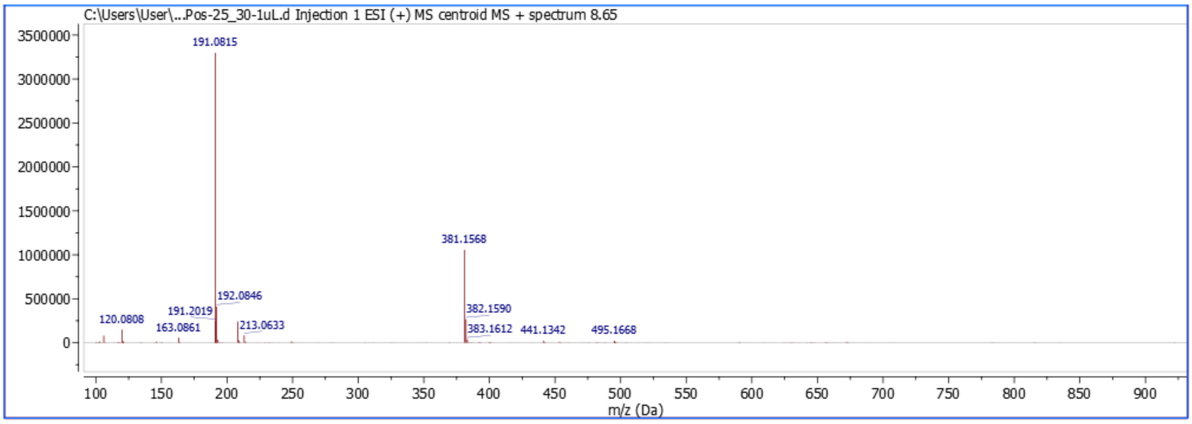

HRMS spectrum of (*S*)-5-benzylimidazolidine-2,4-dione (**H2a**)

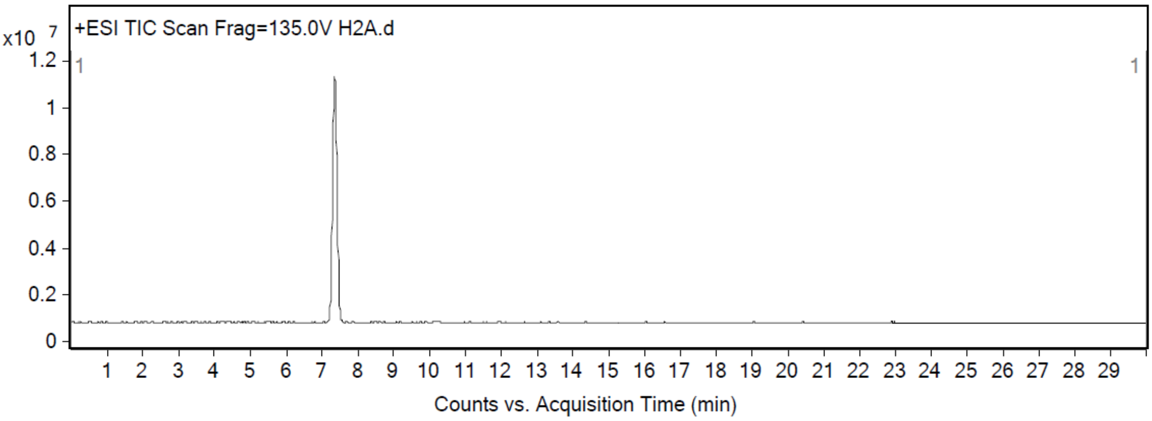

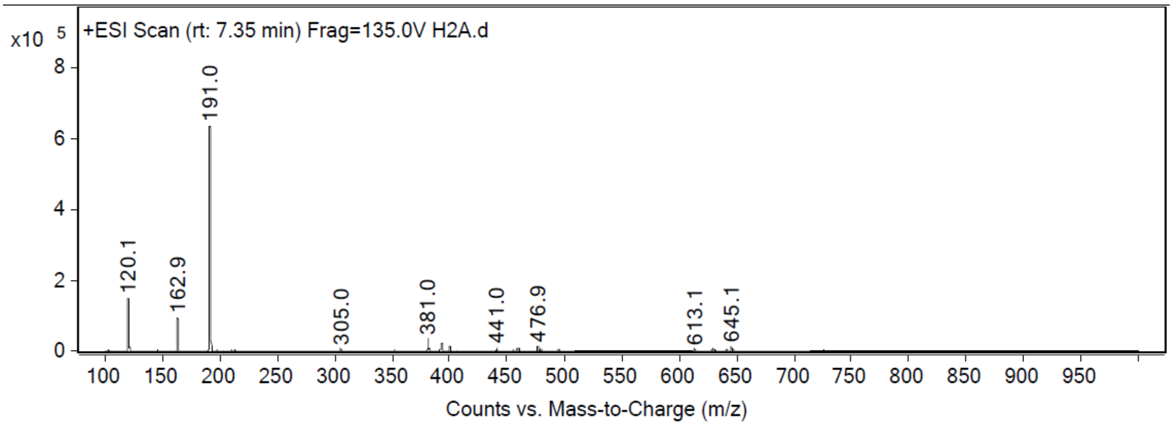

Mass spectrum of (*S*)-5-benzylimidazolidine-2,4-dione (**H2a**)

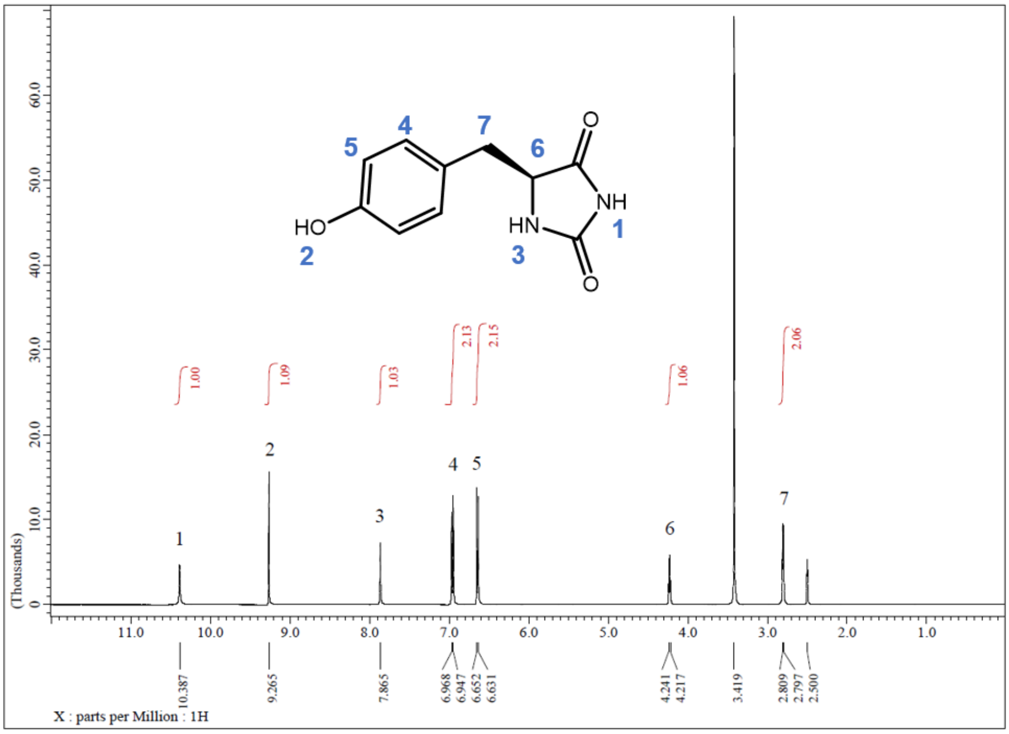

^{1}H NMR spectrum of (*S*)-5-(4-hydroxybenzyl)imidazolidine-2,4-dione (**H2b**)

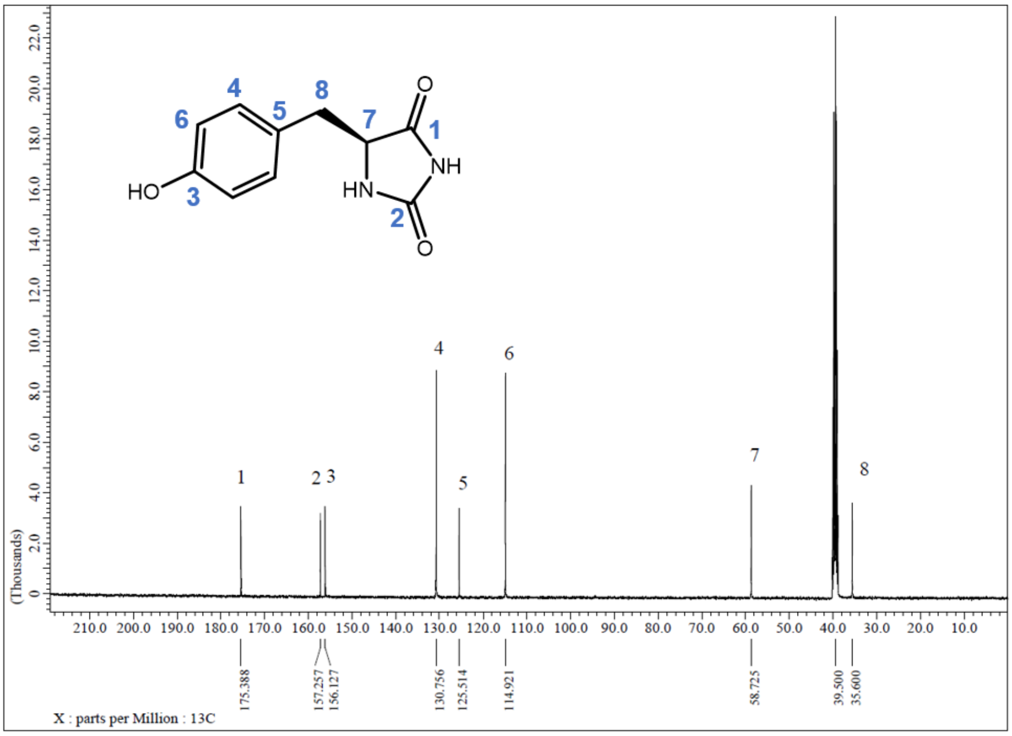

^{13}C NMR spectrum of (*S*)-5-(4-hydroxybenzyl)imidazolidine-2,4-dione (**H2b**)

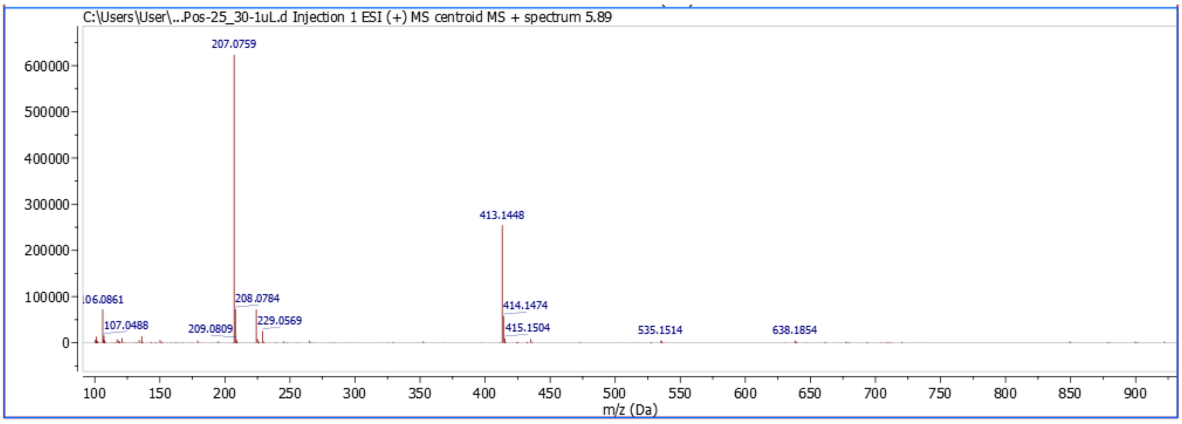

HRMS spectrum of (*S*)-5-(4-hydroxybenzyl)imidazolidine-2,4-dione (**H2b**)

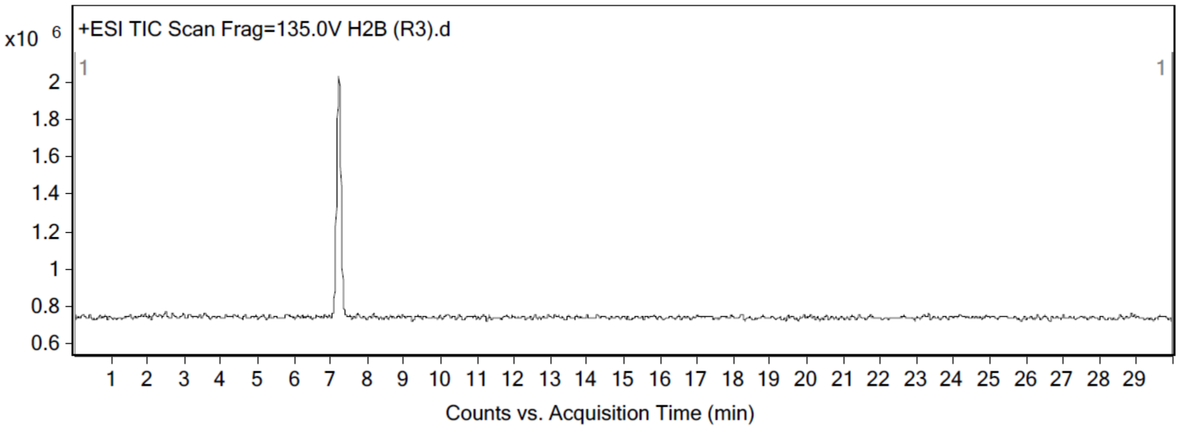

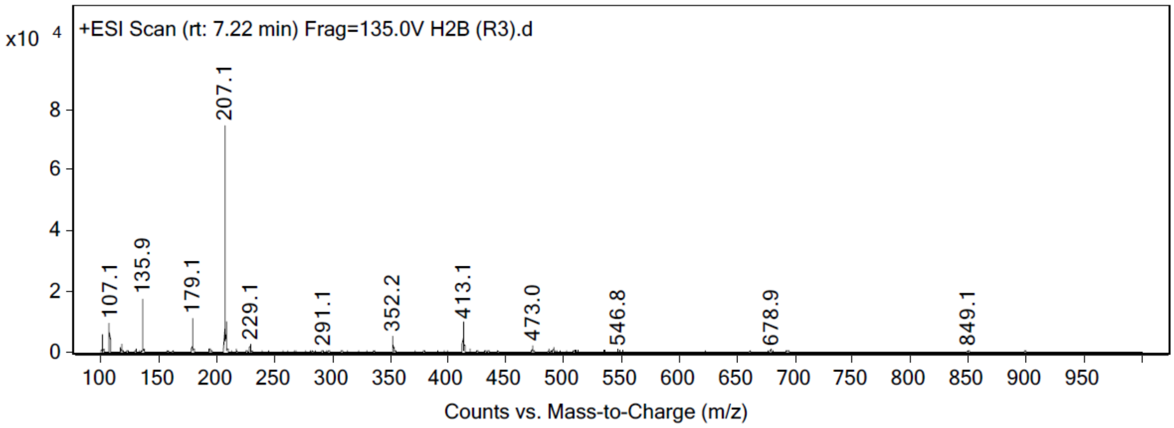

Mass spectrum of (*S*)-5-(4-hydroxybenzyl)imidazolidine-2,4-dione (**H2b**)

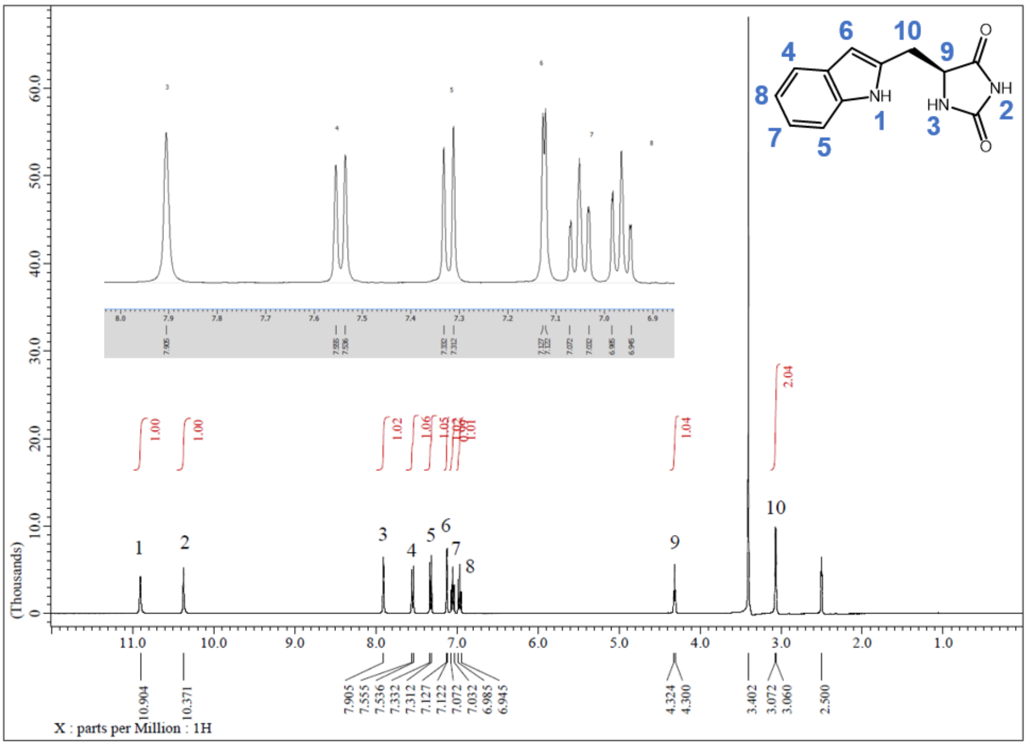

^{1}H NMR spectrum of (*S*)-5-((1*H*-indol-2-yl)methyl)imidazolidine-2,4-dione (**H2c**)

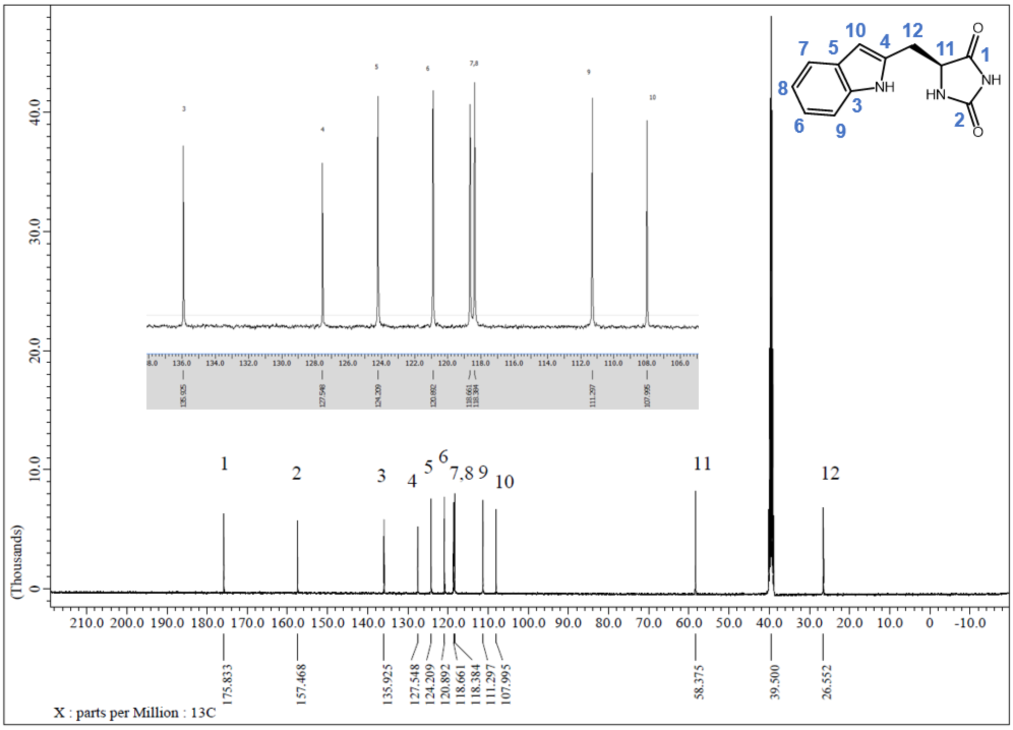

^{13}C NMR spectrum of (*S*)-5-((1*H*-indol-2-yl)methyl)imidazolidine-2,4-dione (**H2c**)

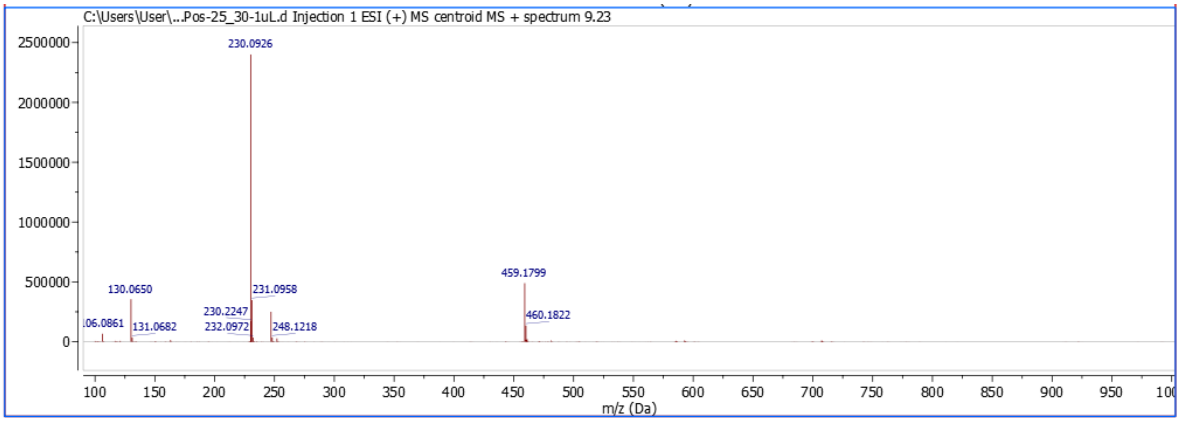

HRMS spectrum of (*S*)-5-((1*H*-indol-2-yl)methyl)imidazolidine-2,4-dione (**H2c**)

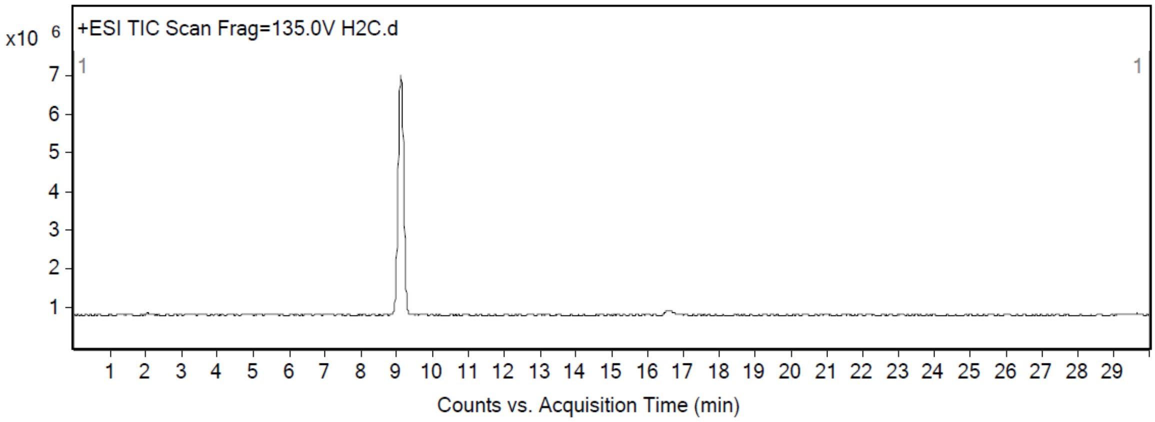

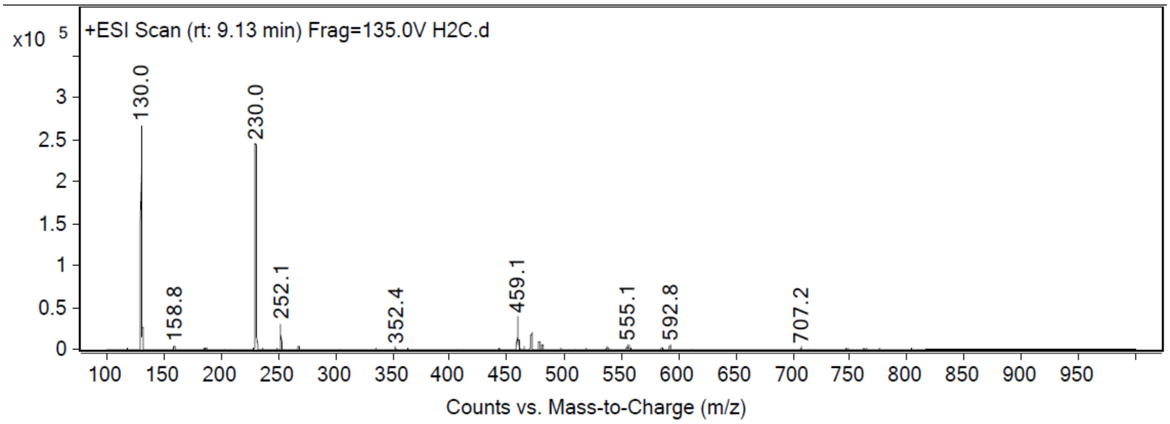

Mass spectrum of (*S*)-5-((1*H*-indol-2-yl)methyl)imidazolidine-2,4-dione (**H2c**)

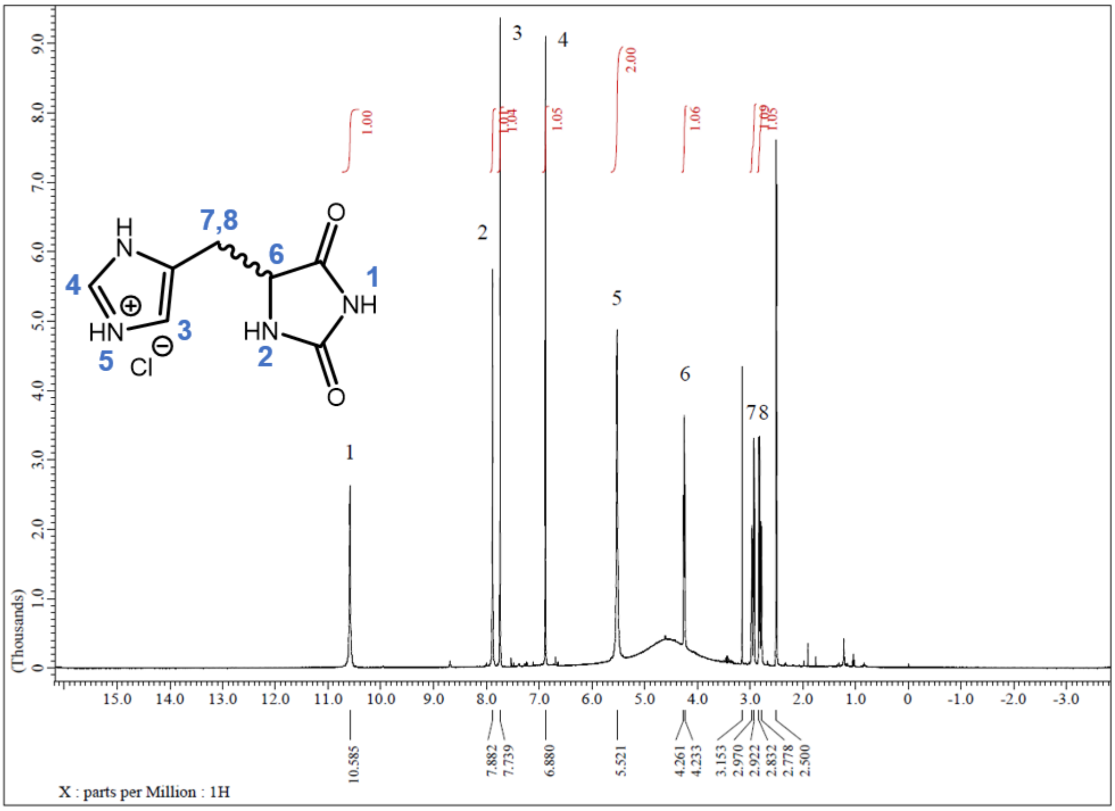

^{1}H NMR spectrum of (*S*)-5-((2,5-dioxoimidazolidin-4-yl)methyl)-1*H*-imidazol-3-ium chloride (**H2d**)

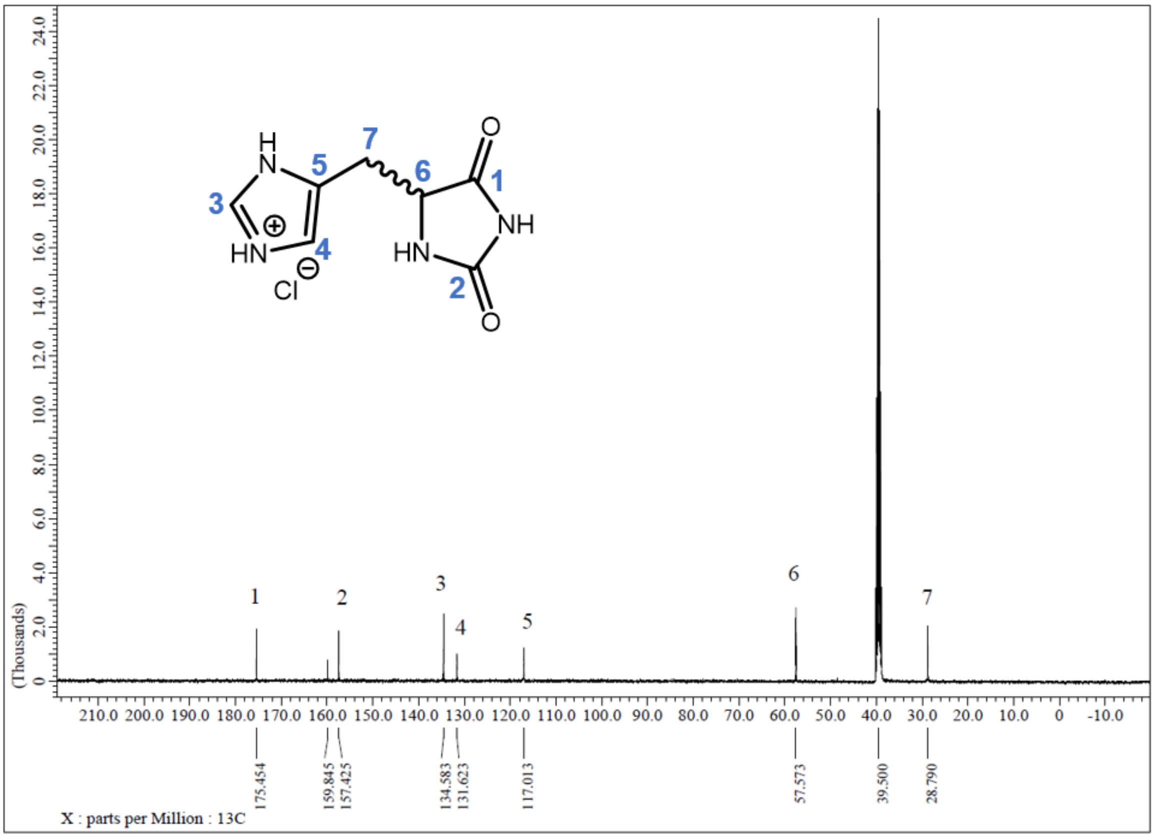

^{13}C NMR spectrum of (*S*)-5-((2,5-dioxoimidazolidin-4-yl)methyl)-1*H*-imidazol-3-ium chloride (**H2d**)

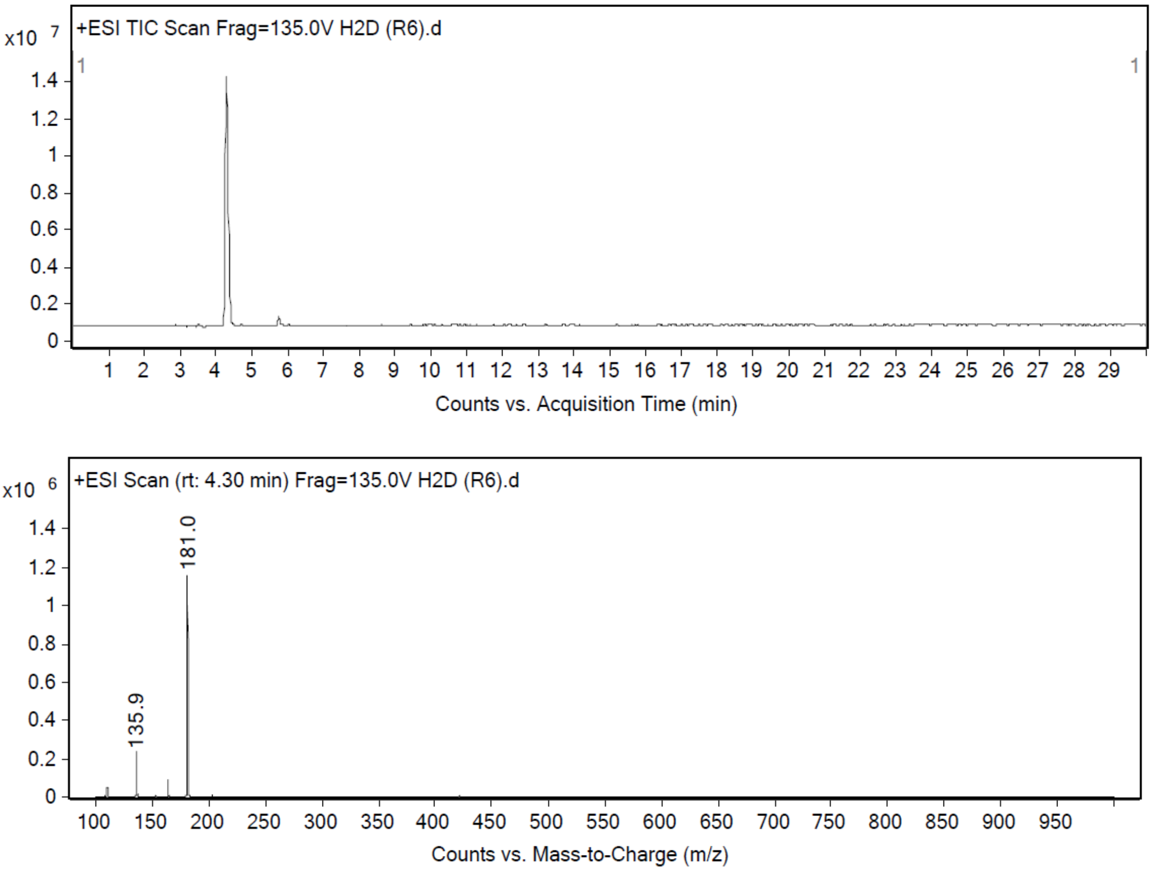

Mass spectrum of (*S*)-5-((2,5-dioxoimidazolidin-4-yl)methyl)-1*H*-imidazol-3-ium chloride (**H2d**)

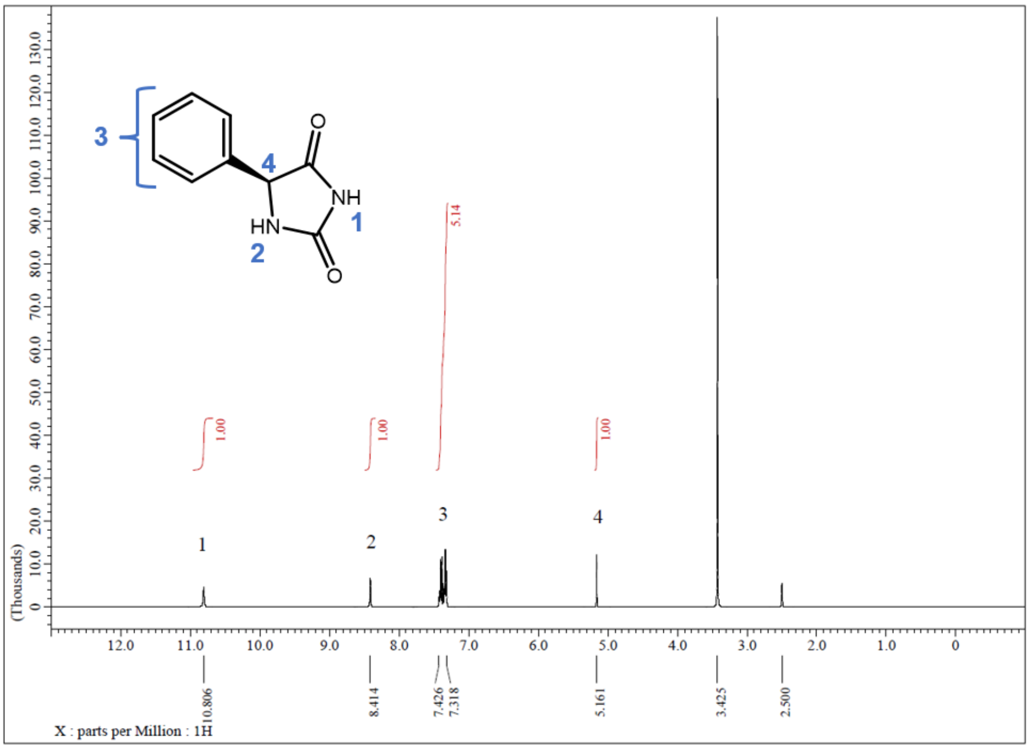

^{1}H NMR spectrum of (*S*)-5-phenylimidazolidine-2,4-dione (**H2e**)

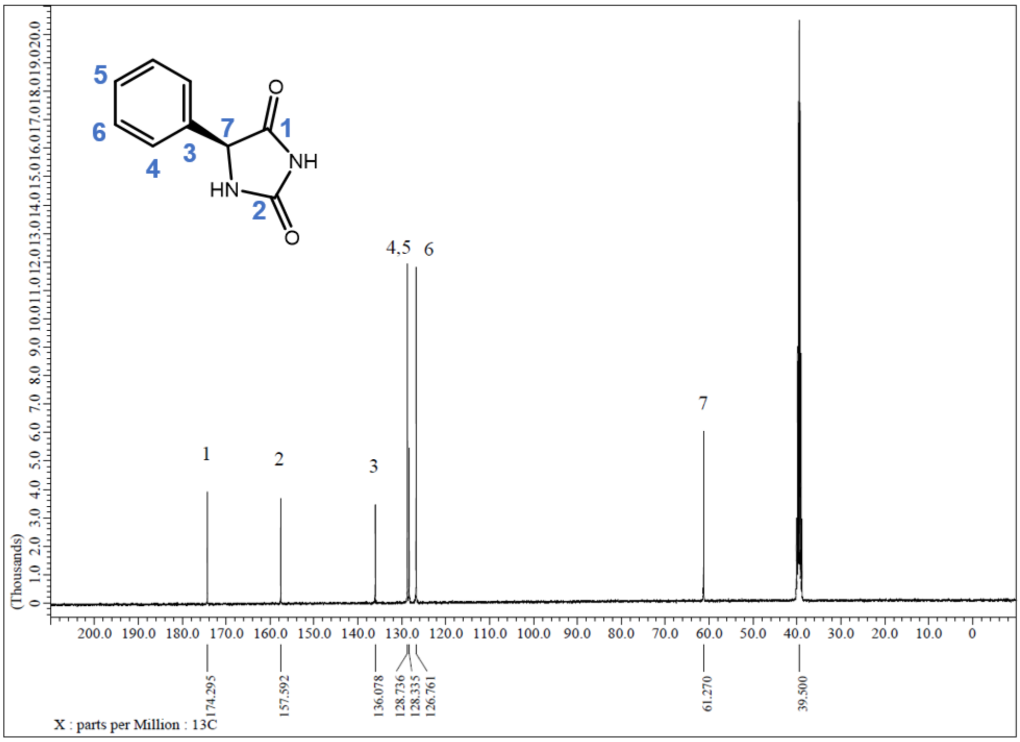

^{13}C NMR spectrum of (*S*)-5-phenylimidazolidine-2,4-dione (**H2e**)

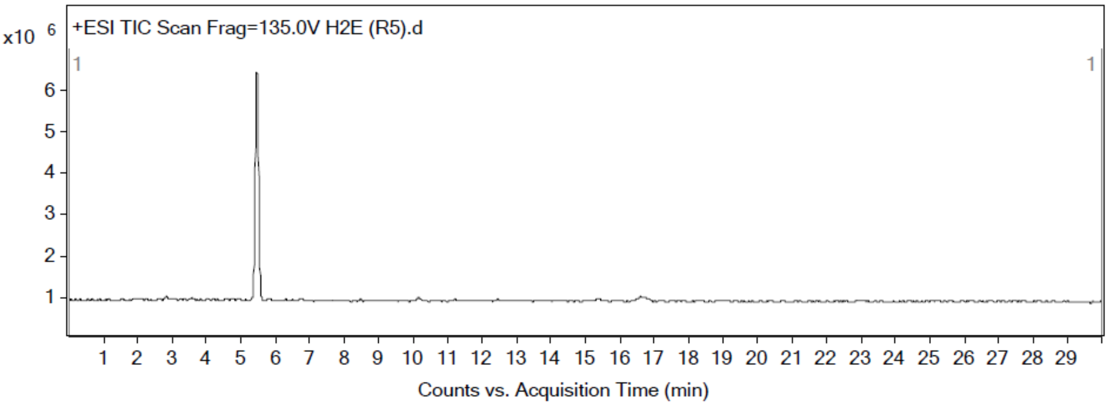

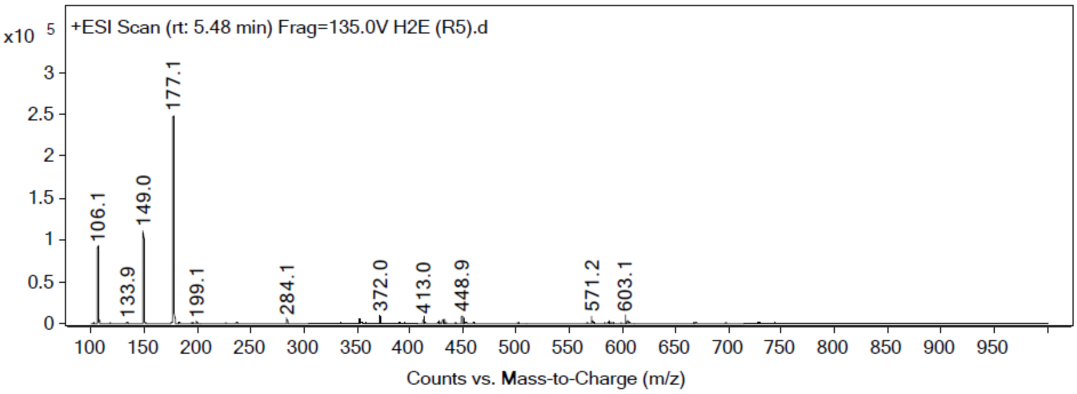

Mass spectrum of (*S*)-5-phenylimidazolidine-2,4-dione (**H2e**)

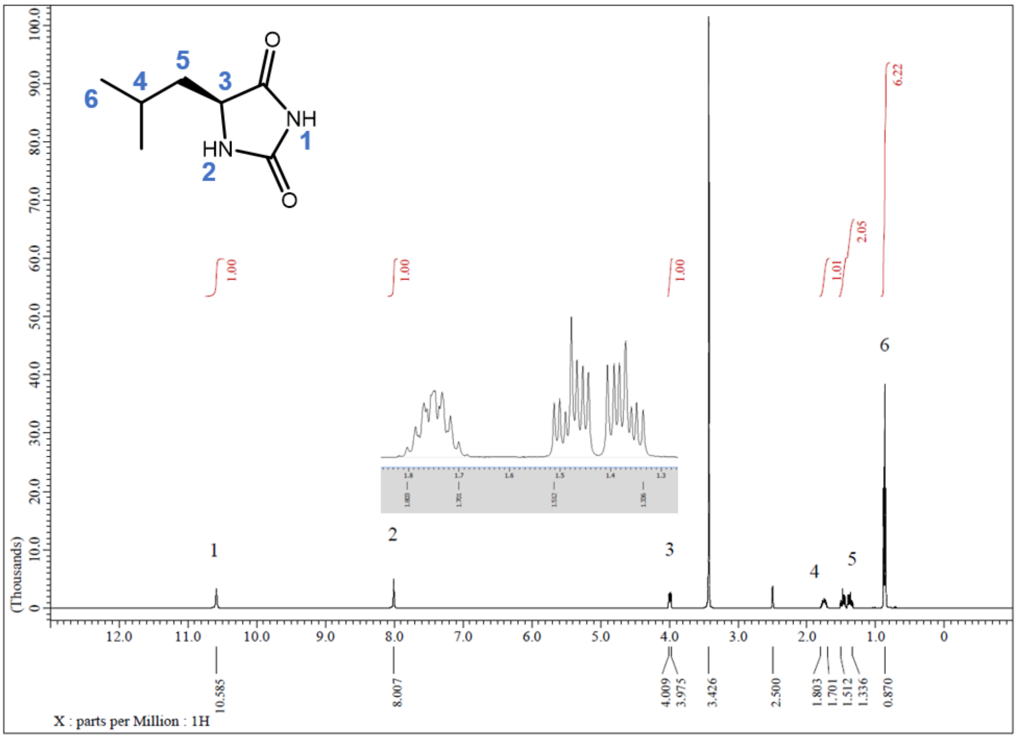

^{1}H NMR spectrum of (*S*)-5-isobutylimidazolidine-2,4-dione (**H2f**)

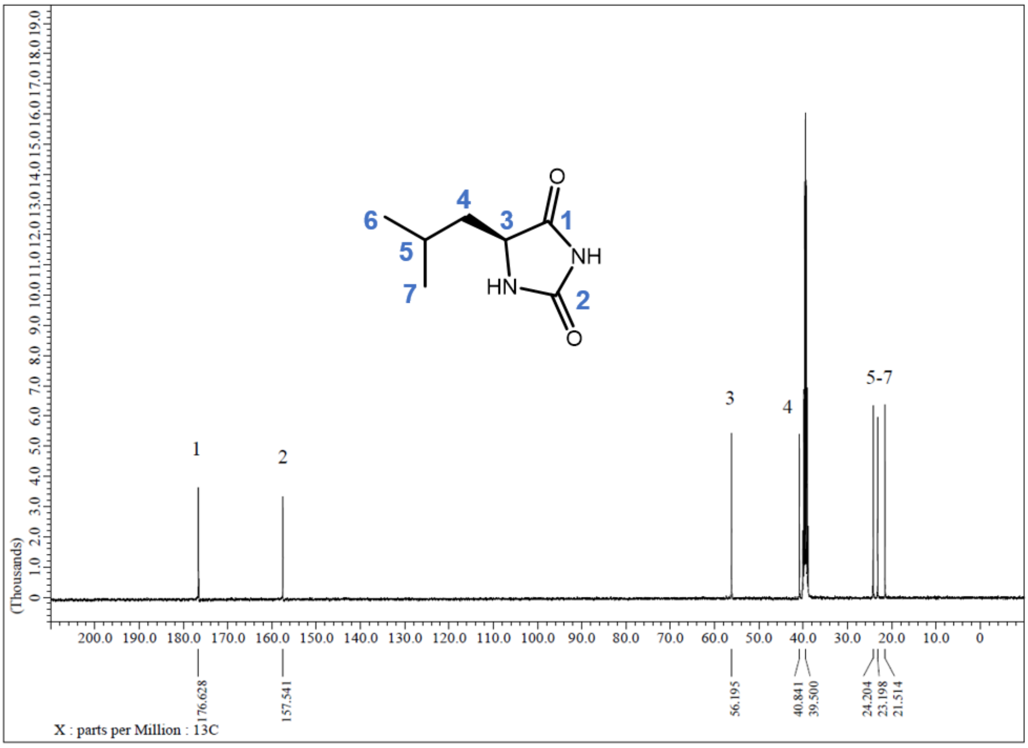

^{13}C NMR spectrum of (*S*)-5-isobutylimidazolidine-2,4-dione (**H2f**)

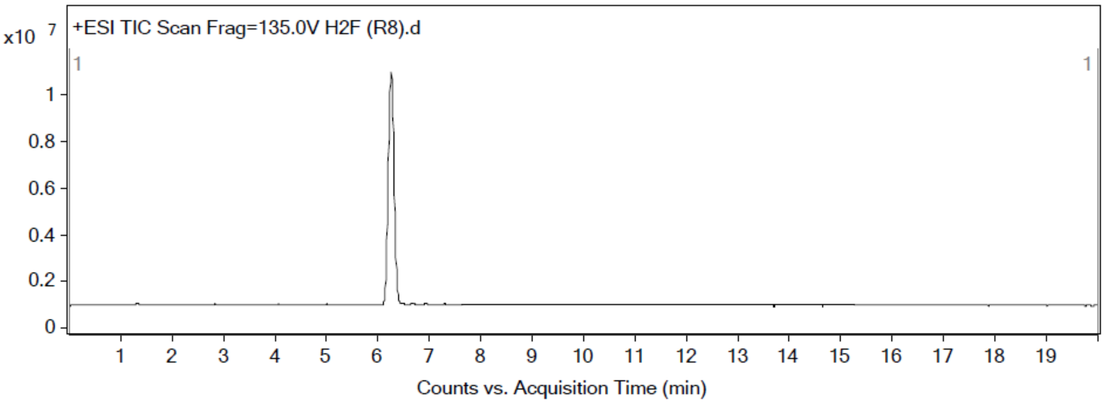

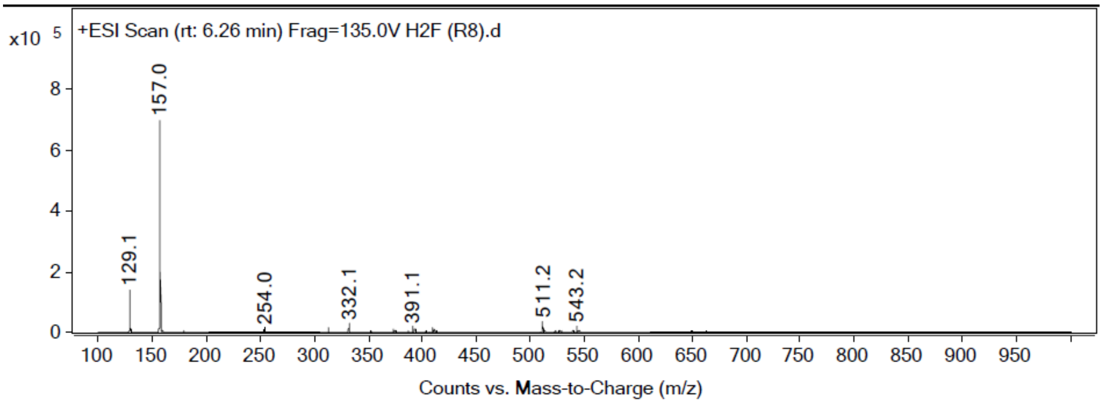

Mass spectrum of (*S*)-5-isobutylimidazolidine-2,4-dione (**H2f**)

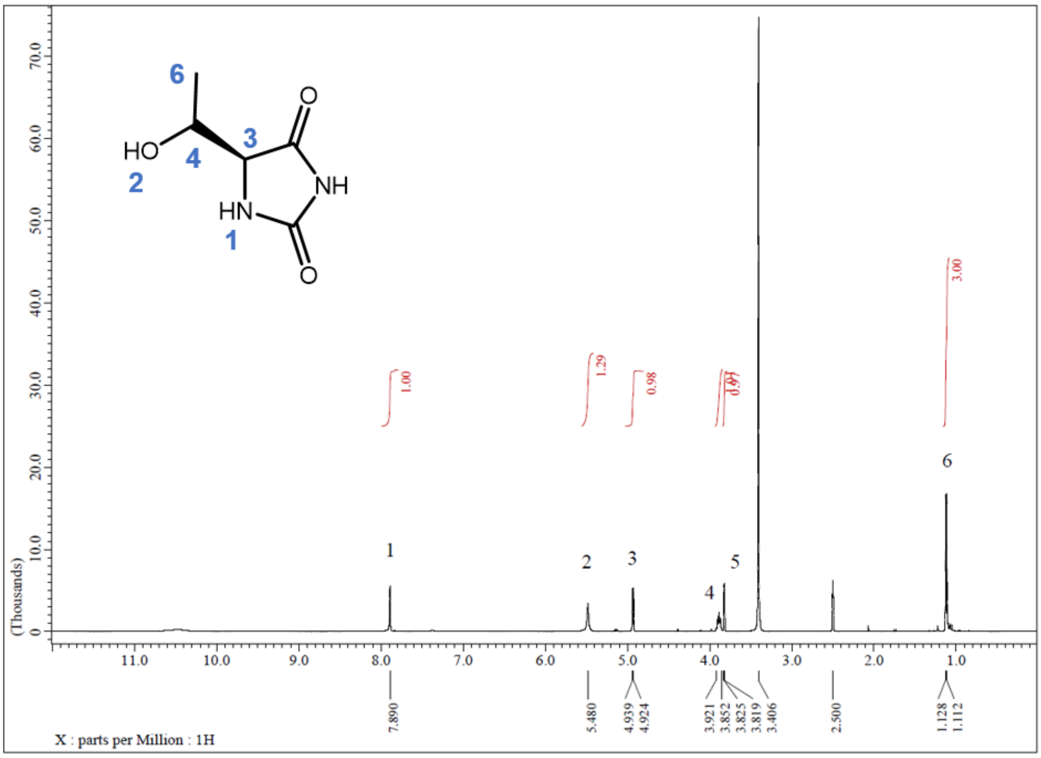

^{1}H NMR spectrum of (*S*)-5-((*R*)-1-hydroxyethyl)imidazolidine-2,4-dione (**H2g**)

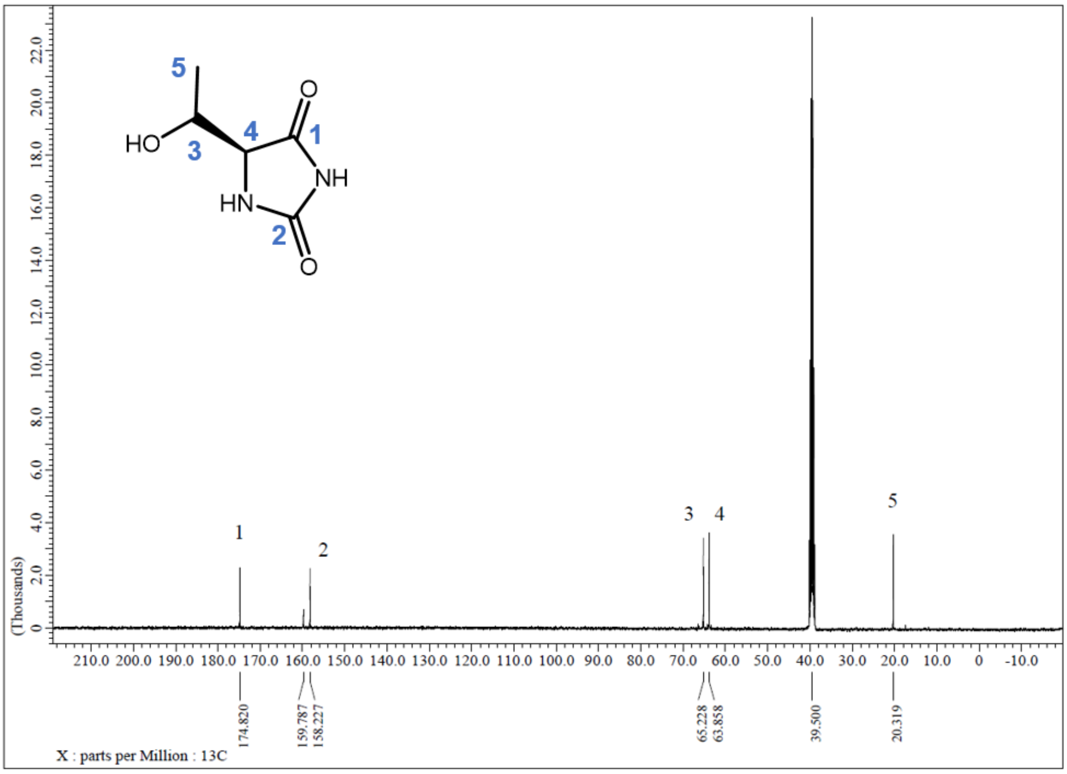

^{13}C NMR spectrum of (*S*)-5-((*R*)-1-hydroxyethyl)imidazolidine-2,4-dione (**H2g**)

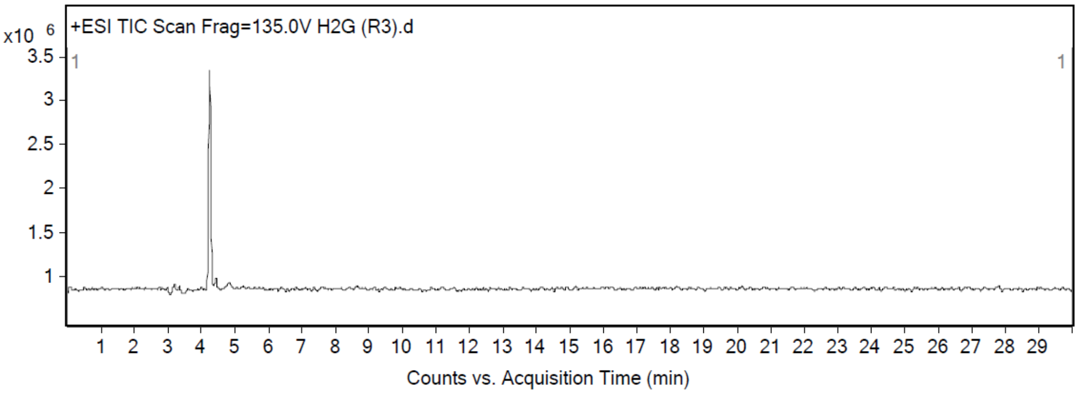

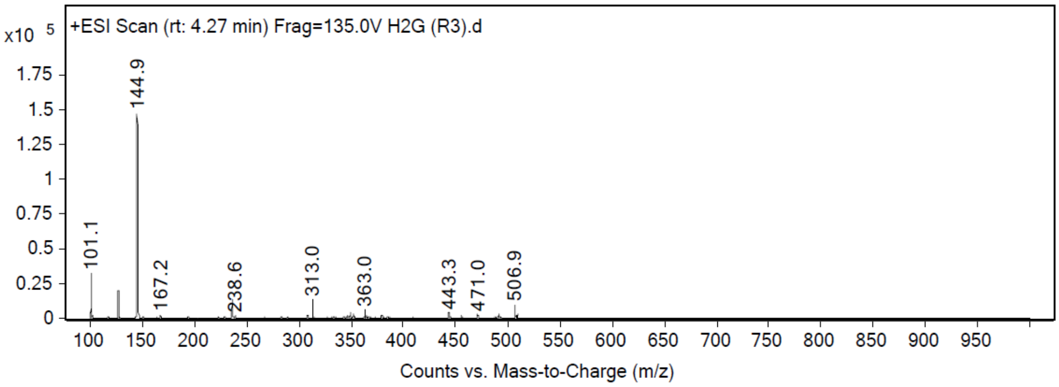

Mass spectrum of (*S*)-5-((*R*)-1-hydroxyethyl)imidazolidine-2,4-dione (**H2g**)

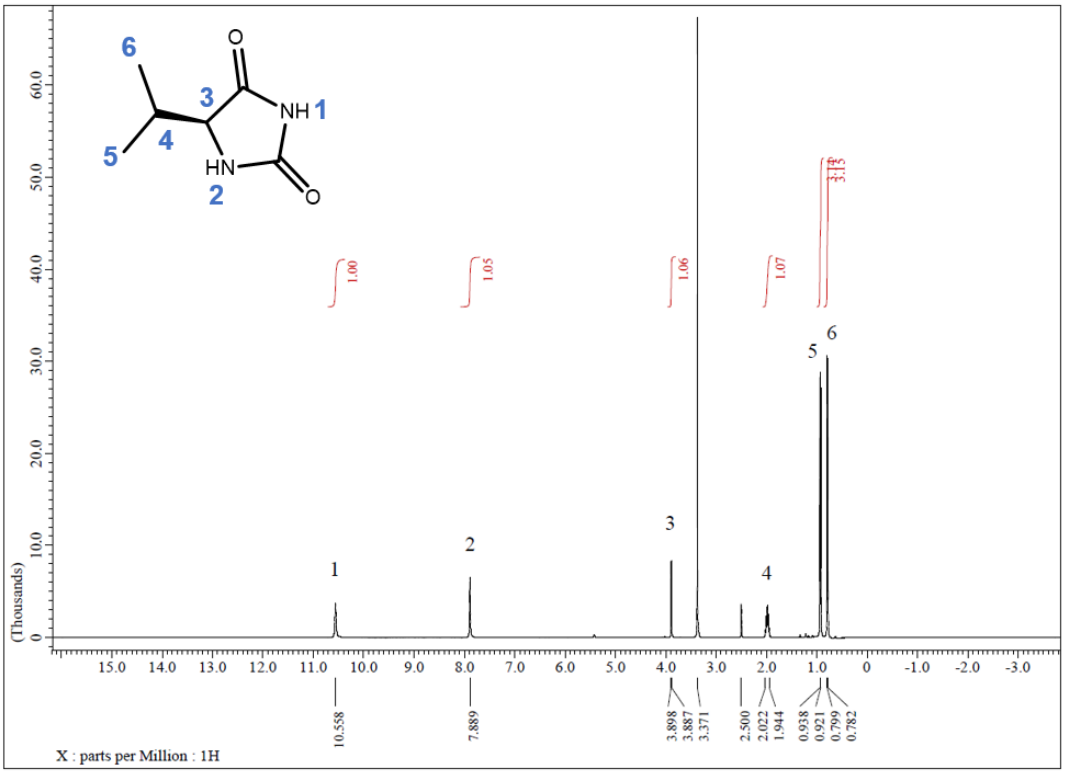

^{1}H NMR spectrum of (*S*)-5-isopropylimidazolidine-2,4-dione (**H2h**)

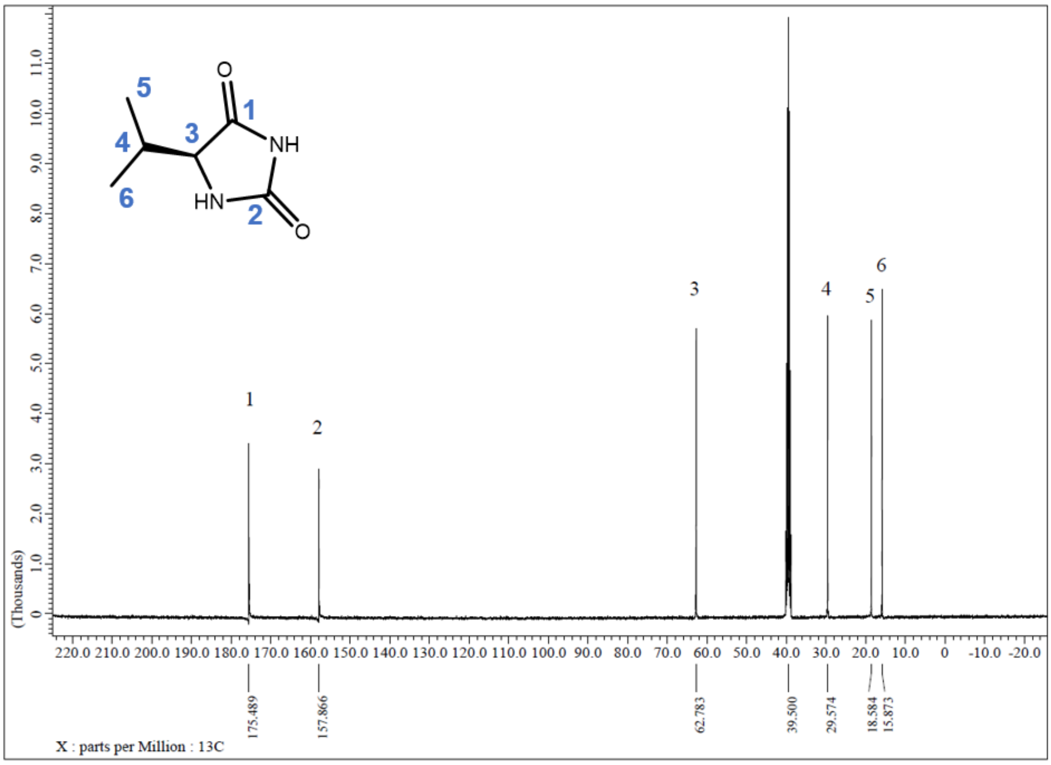

^{13}C NMR spectrum of (*S*)-5-isopropylimidazolidine-2,4-dione (**H2h**)

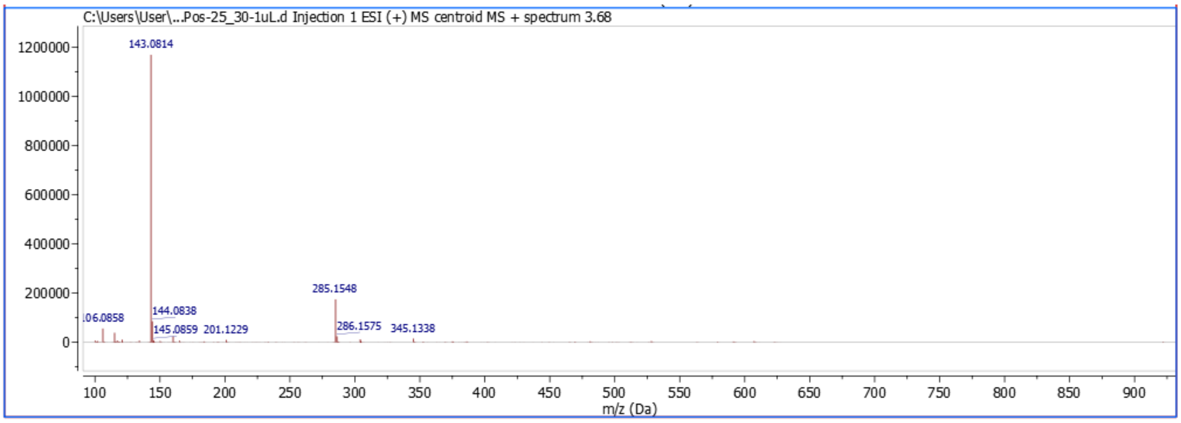

HRMS spectrum of (*S*)-5-isopropylimidazolidine-2,4-dione (**H2h**)

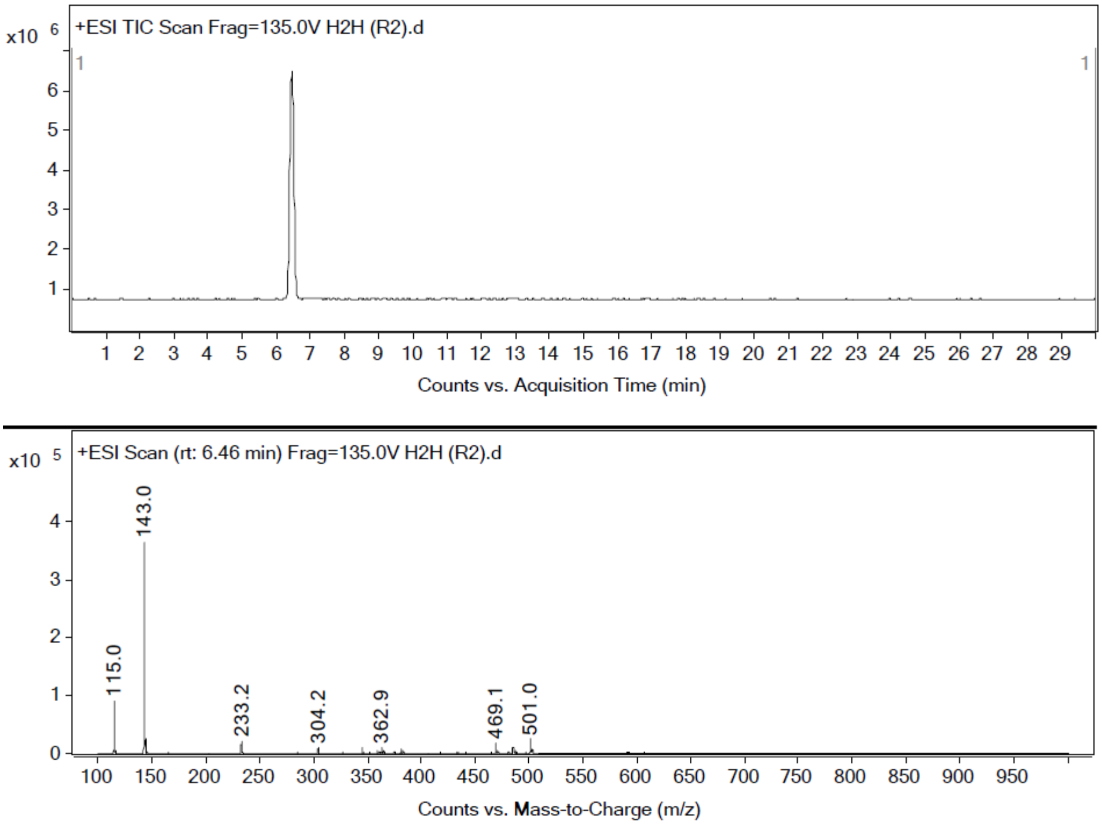

Mass spectrum of (*S*)-5-isopropylimidazolidine-2,4-dione (**H2h**)

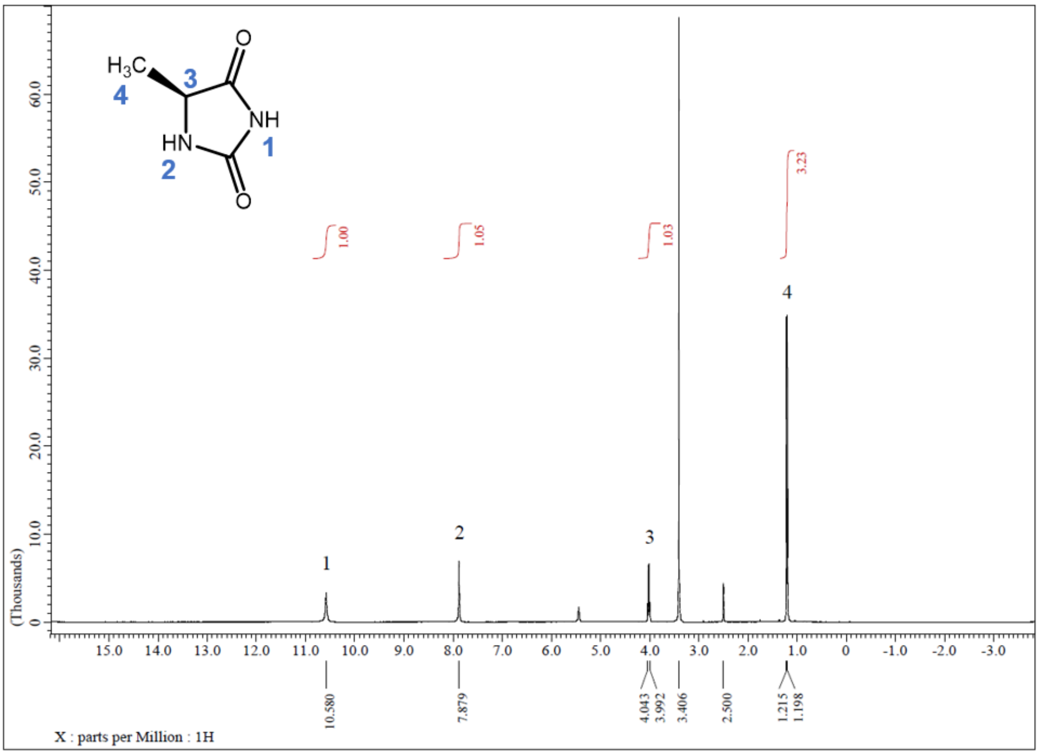

^{1}H NMR spectrum of (*S*)-5-methylimidazolidine-2,4-dione (**H2i**)

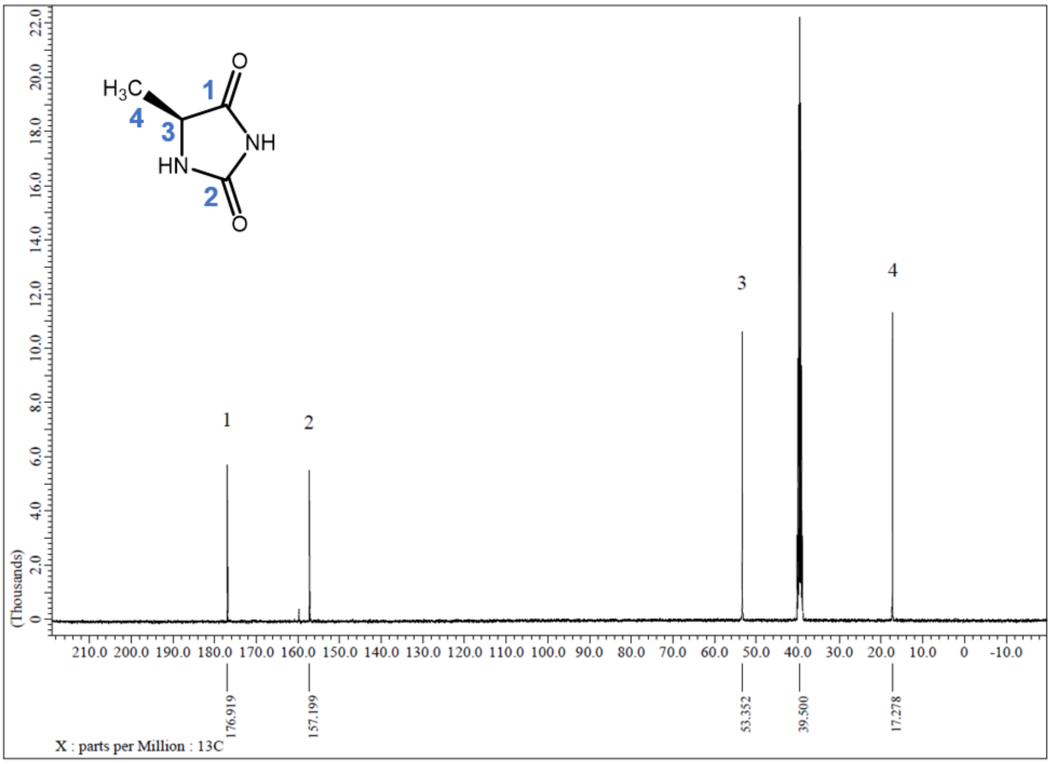

^{13}C NMR spectrum of (*S*)-5-methylimidazolidine-2,4-dione (**H2i**)

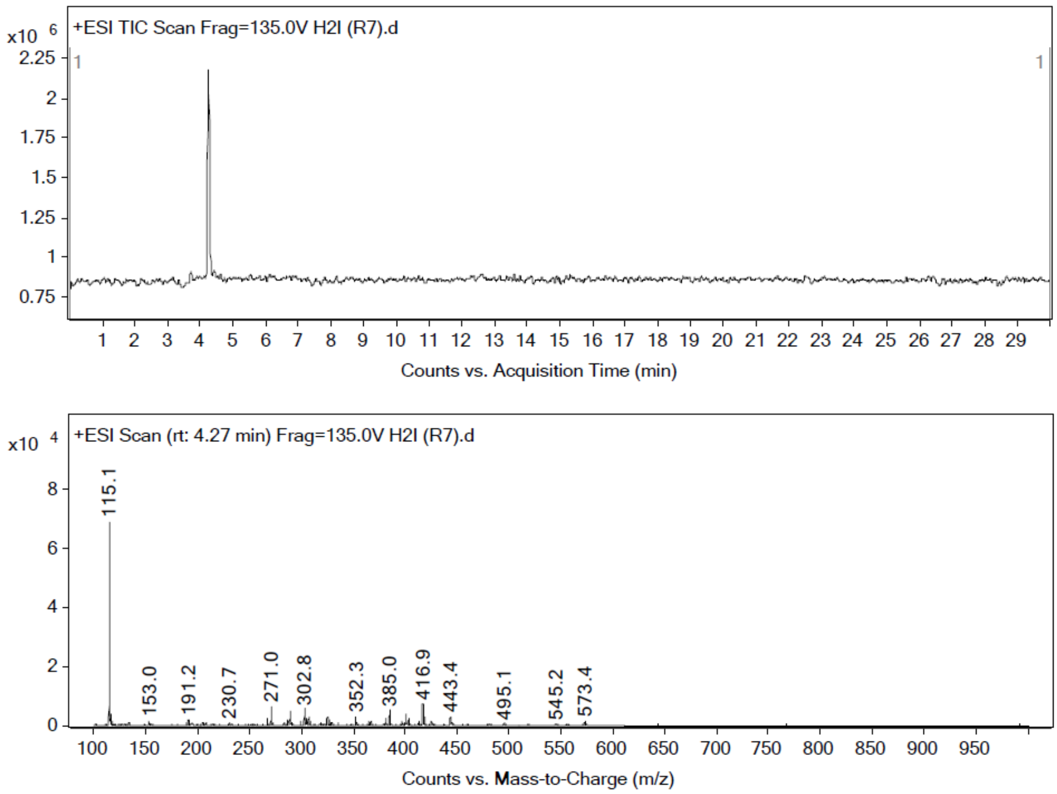

Mass spectrum of (*S*)-5-methylimidazolidine-2,4-dione (**H2i**)

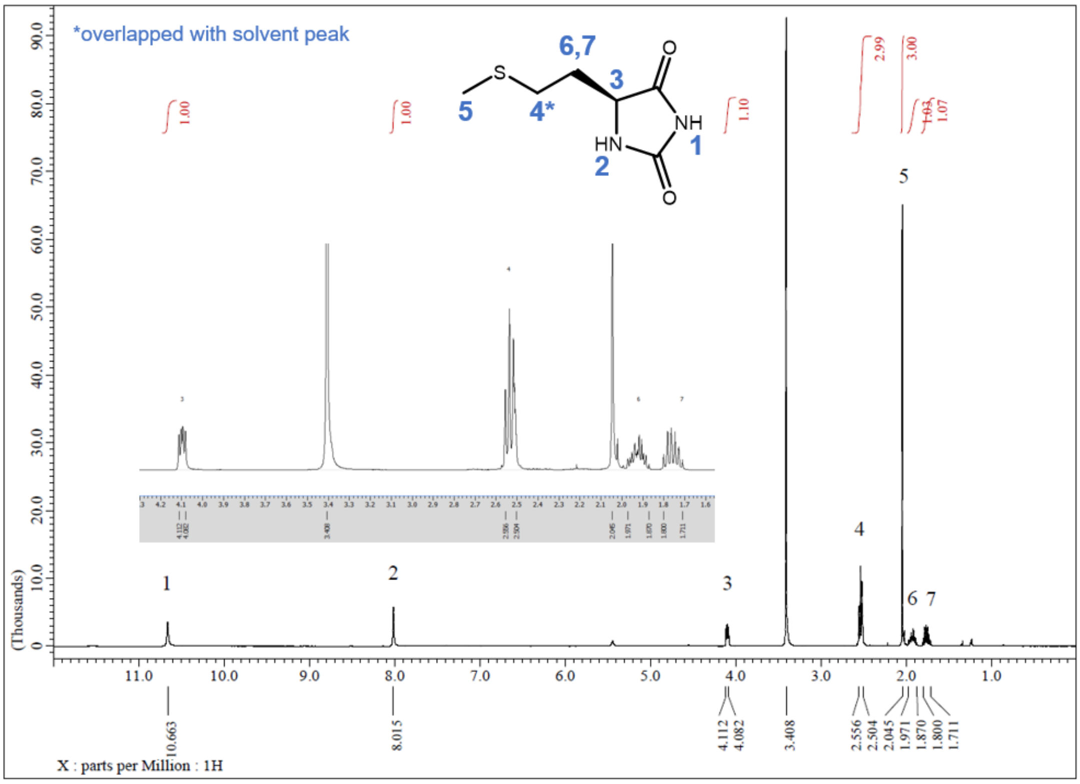

^{1}H NMR spectrum of (*S*)-5-(2-(methylthio)ethyl)imidazolidine-2,4-dione (**H2j**)

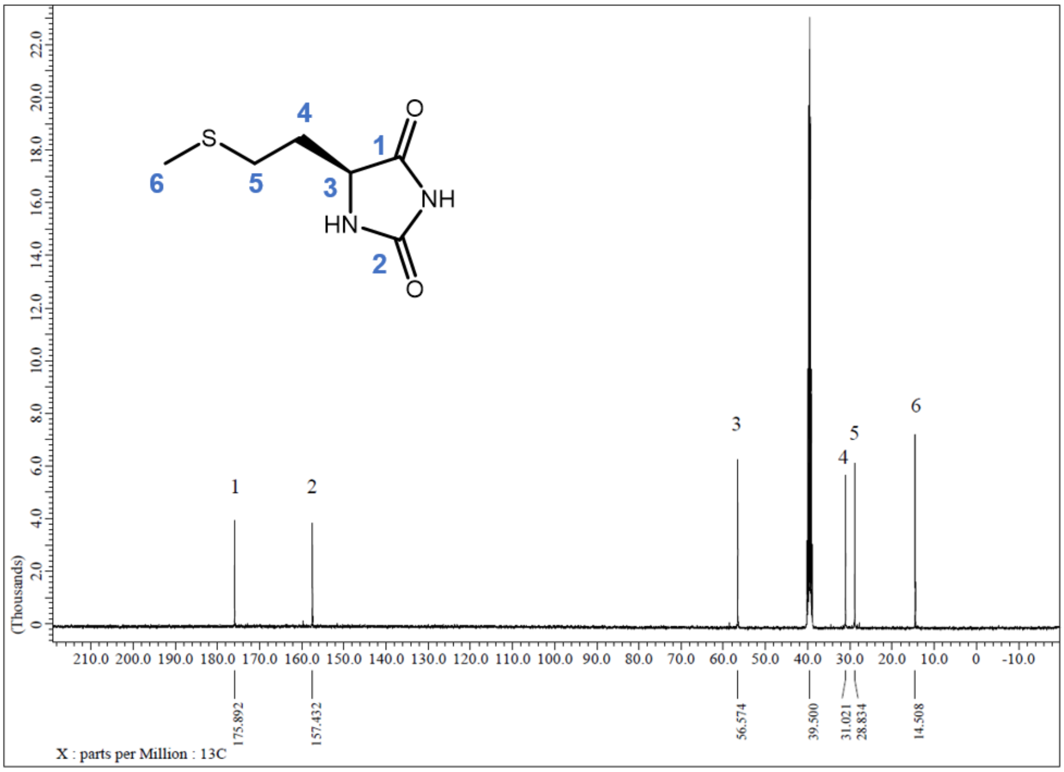

^{13}C NMR spectrum of (*S*)-5-(2-(methylthio)ethyl)imidazolidine-2,4-dione (**H2j**)

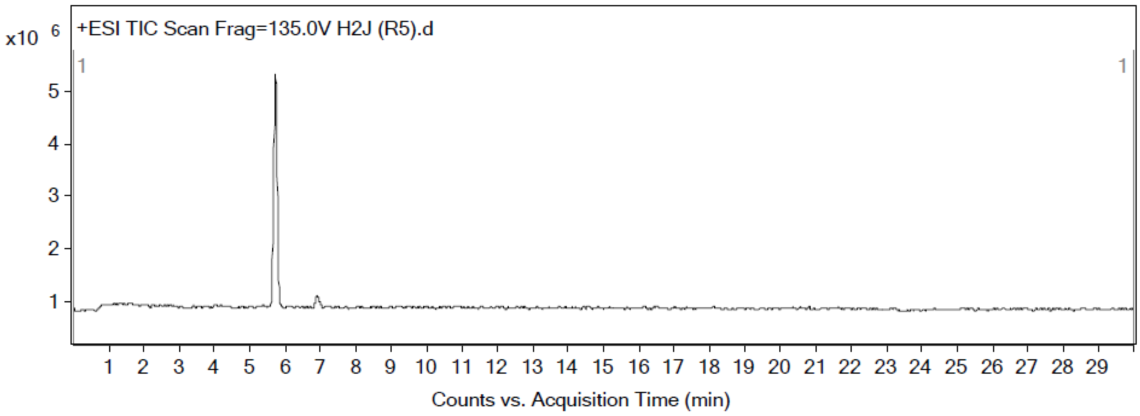

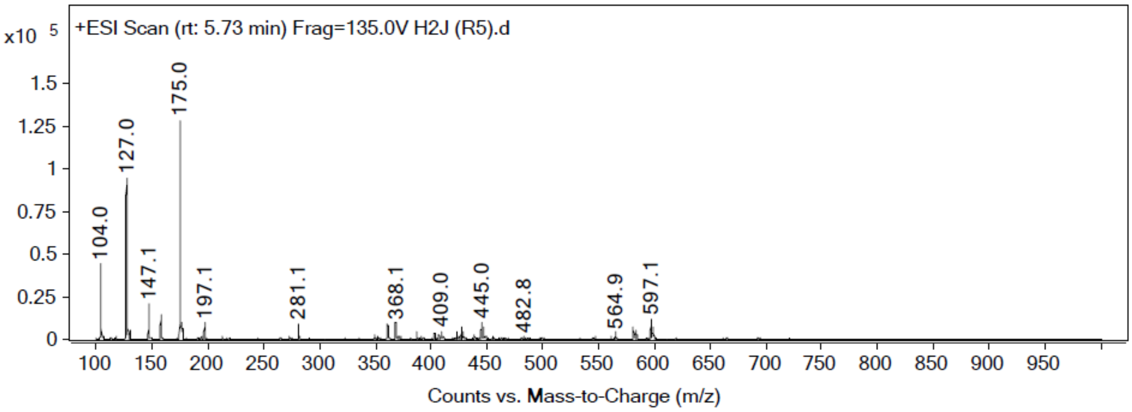

Mass spectrum of (*S*)-5-(2-(methylthio)ethyl)imidazolidine-2,4-dione (**H2j**)

4. Chromatograms of compounds

```
Acq. Operator   : SYSTEM                          Seq. Line :   1
Acq. Instrument : HPLC 1260                        Location : Vial 1
Injection Date  : 4/7/2024 3:32:06 PM                   Inj :   1
                                                 Inj Volume : 10.000 µl
Method          : C:\CHEM32\1\DATA\CHANG WEI JIN H2A 2024-07-04 15-30-36\HYDANTOIN.M (
                  Sequence Method)
Last changed    : 4/7/2024 3:30:45 PM by SYSTEM
```

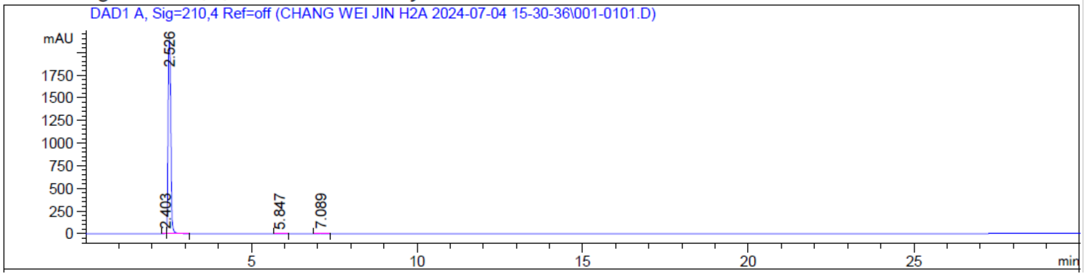

Signal 1: DAD1 A, Sig=210,4 Ref=off

Peak #	RetTime [min]	Type	Width [min]	Area [mAU*s]	Height [mAU]	Area %
1	2.403	BV	0.0480	20.58212	6.67029	0.1715
2	2.526	VB	0.0913	1.19494e4	2133.56299	99.5478
3	5.847	BB	0.1119	7.91664	1.07548	0.0660
4	7.089	BB	0.1312	25.78545	3.03190	0.2148
Totals :				1.20037e4	2144.34066	

Chromatogram and HPLC integrations of **H2a**.

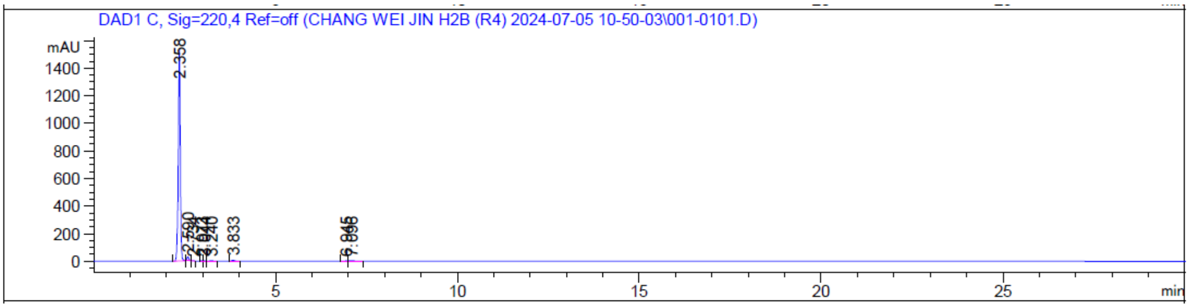

Signal 3: DAD1 C, Sig=220,4 Ref=off

Peak #	RetTime [min]	Type	Width [min]	Area [mAU*s]	Height [mAU]	Area %
1	2.358	BV	0.0581	5826.54150	1543.05322	95.9929
2	2.590	VB	0.0581	118.87553	31.47240	1.9585
3	2.734	BB	0.0524	5.85003	1.77786	0.0964
4	2.973	BV	0.0584	9.89534	2.59918	0.1630
5	3.044	VV	0.0557	5.55367	1.48331	0.0915
6	3.240	VB	0.1110	14.20711	1.95022	0.2341
7	3.833	BB	0.0822	37.24251	6.98077	0.6136
8	6.945	BV	0.1061	15.36413	2.18387	0.2531
9	7.096	VB	0.1371	36.23384	3.94834	0.5970
Totals :				6069.76367	1595.44917	

Chromatogram and HPLC integrations of **H2b**.

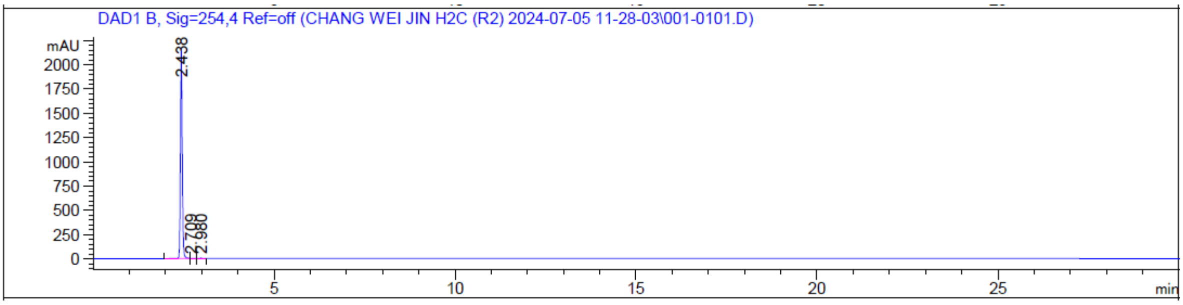

Signal 2: DAD1 B, Sig=254,4 Ref=off

Peak #	RetTime [min]	Type	Width [min]	Area [mAU*s]	Height [mAU]	Area %
1	2.438	BV	0.0583	8261.82910	2176.68433	99.6956
2	2.709	VB	0.0679	9.51642	2.05883	0.1148
3	2.980	BB	0.0703	15.70722	3.37788	0.1895
Totals :				8287.05273	2182.12103	

Chromatogram and HPLC integrations of **H2c**.

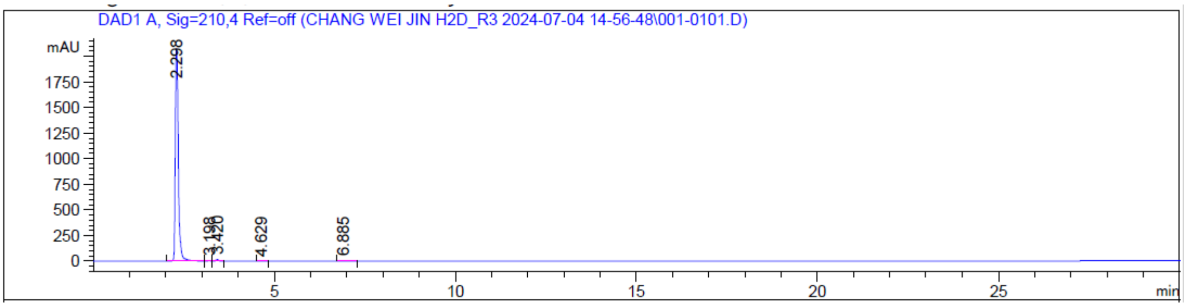

Signal 1: DAD1 A, Sig=210,4 Ref=off

Peak #	RetTime [min]	Type	Width [min]	Area [mAU*s]	Height [mAU]	Area %
1	2.298	BB	0.0909	1.19004e4	2074.88818	99.1798
2	3.198	BV	0.0828	9.44985	1.65101	0.0788
3	3.420	VB	0.0741	65.08628	13.05972	0.5424
4	4.629	BB	0.0908	6.53019	1.10785	0.0544
5	6.885	BB	0.1238	17.34209	2.15695	0.1445
Totals :				1.19988e4	2092.86372	

Chromatogram and HPLC integrations of **H2d**.

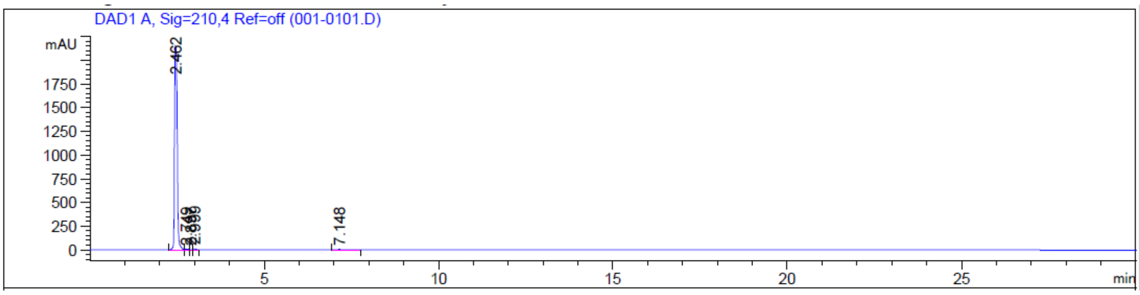

Signal 1: DAD1 A, Sig=210,4 Ref=off

Peak #	RetTime [min]	Type	Width [min]	Area [mAU*s]	Height [mAU]	Area %
1	2.462	BV	0.0866	1.15082e4	2145.84326	98.9389
2	2.749	VV	0.0870	36.11869	5.93891	0.3105
3	2.897	VV	0.0545	7.37558	1.93413	0.0634
4	2.999	VB	0.0673	49.22173	11.20341	0.4232
5	7.148	BB	0.1362	30.71308	3.43865	0.2640
Totals :				1.16317e4	2168.35836	

Chromatogram and HPLC integrations of **H2e**.

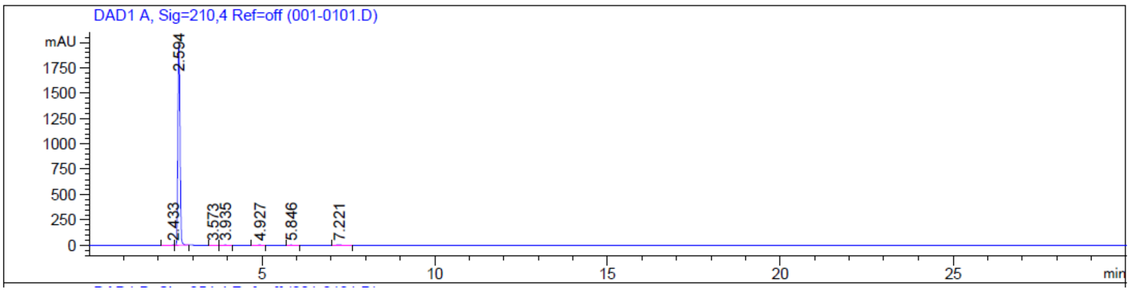

Signal 1: DAD1 A, Sig=210,4 Ref=off

Peak #	RetTime [min]	Type	Width [min]	Area [mAU*s]	Height [mAU]	Area %
1	2.433	BB	0.0740	15.73734	2.96331	0.1829
2	2.594	BB	0.0675	8455.26465	1993.84448	98.2673
3	3.573	VB	0.1246	21.32673	2.33320	0.2479
4	3.935	BB	0.1093	22.54987	3.01346	0.2621
5	4.927	BB	0.1084	21.42057	2.82658	0.2490
6	5.846	BB	0.1085	16.58592	2.40312	0.1928
7	7.221	BB	0.1300	51.46523	6.12673	0.5981
Totals :				8604.35031	2013.51088	

Chromatogram and HPLC integrations of **H2f**.

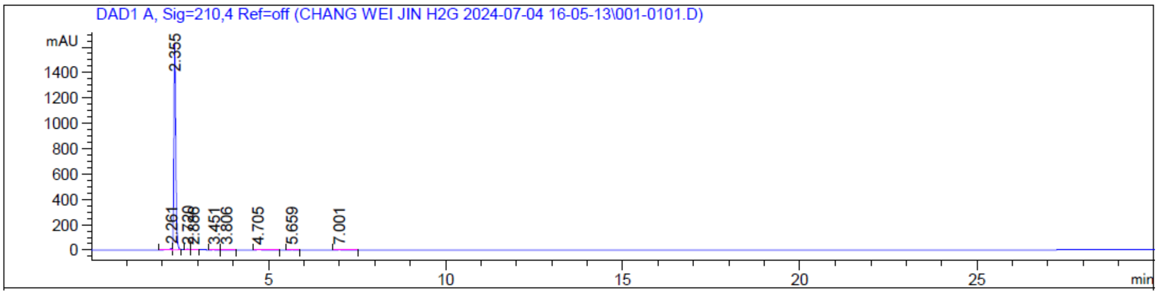

Signal 1: DAD1 A, Sig=210,4 Ref=off

Peak #	RetTime [min]	Type	Width [min]	Area [mAU*s]	Height [mAU]	Area %
1	2.261	BV	0.0692	55.54348	11.03689	0.8953
2	2.355	VV	0.0588	6015.70117	1639.38928	96.9619
3	2.720	BV	0.0812	16.12285	2.97426	0.2599
4	2.886	VB	0.0849	9.75303	1.65237	0.1572
5	3.451	BB	0.1131	13.81607	1.69416	0.2227
6	3.806	BB	0.1069	15.91802	2.13667	0.2566
7	4.705	BB	0.1313	36.14483	3.65674	0.5826
8	5.659	BB	0.1050	16.47449	2.43191	0.2655
9	7.001	BB	0.1359	24.71371	2.77591	0.3983
Totals :				6204.18764	1667.74819	

Chromatogram and HPLC integrations of **H2g**.

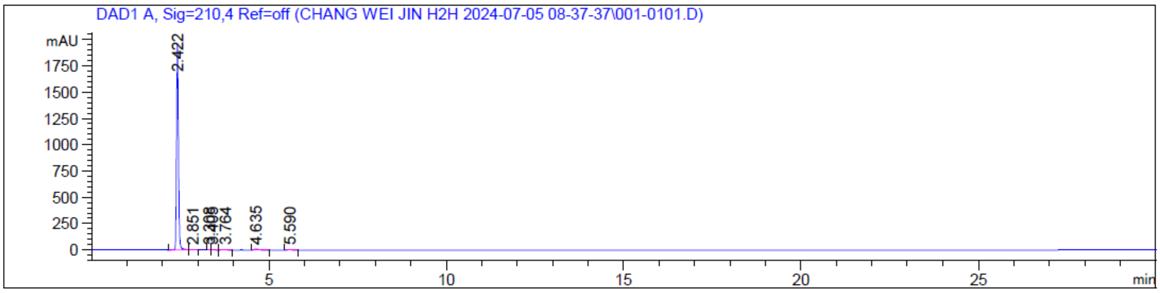

Signal 1: DAD1 A, Sig=210,4 Ref=off

Peak #	RetTime [min]	Type	Width [min]	Area [mAU*s]	Height [mAU]	Area %
1	2.422	BV	0.0646	7871.98828	1968.86609	98.3380
2	2.851	VB	0.1019	23.49626	3.19237	0.2935
3	3.308	BV	0.0706	8.47418	1.81192	0.1059
4	3.405	VB	0.0874	11.54817	1.88816	0.1443
5	3.764	BB	0.1185	15.21869	1.80299	0.1901
6	4.635	BB	0.1022	61.07473	8.66549	0.7630
7	5.590	BB	0.1048	13.23519	1.96022	0.1653

Totals : 8005.03551 1988.18724

Chromatogram and HPLC integrations of **H2h**.

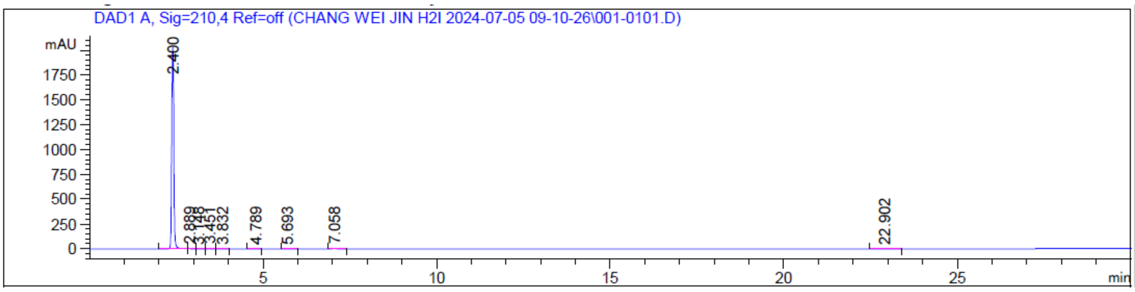

Signal 1: DAD1 A, Sig=210,4 Ref=off

Peak #	RetTime [min]	Type	Width [min]	Area [mAU*s]	Height [mAU]	Area %
1	2.400	BV	0.0699	8741.37305	2044.75537	97.5539
2	2.889	VV	0.1088	30.63241	3.76871	0.3419
3	3.148	VV	0.1633	28.19417	2.23343	0.3146
4	3.451	VB	0.1410	32.44205	3.07816	0.3621
5	3.832	BB	0.1322	16.16709	1.71199	0.1804
6	4.789	BB	0.1154	16.98713	2.07756	0.1896
7	5.693	BB	0.1082	14.48612	2.05597	0.1617
8	7.058	BB	0.1308	49.59040	5.97726	0.5534
9	22.902	BB	0.3420	30.68933	1.32613	0.3425

Totals : 8960.56175 2066.98460

Chromatogram and HPLC integrations of **H2i**.

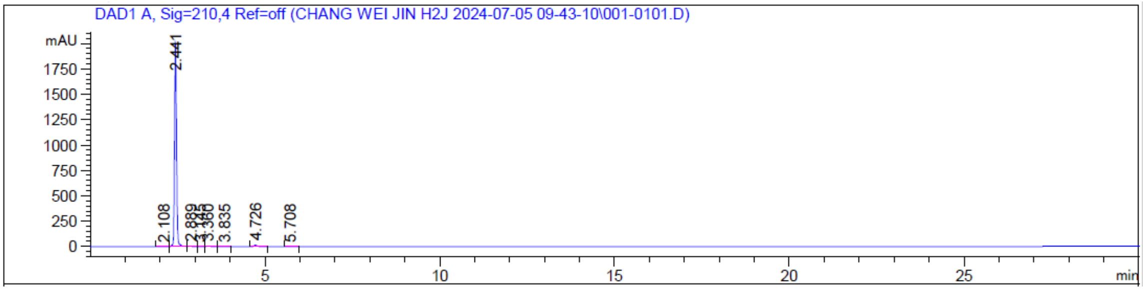

Signal 1: DAD1 A, Sig=210,4 Ref=off

Peak #	RetTime [min]	Type	Width [min]	Area [mAU*s]	Height [mAU]	Area %
1	2.108	BV	0.2010	21.04398	1.64822	0.2402
2	2.441	VV	0.0691	8506.89258	2020.25354	97.0935
3	2.889	VV	0.1384	45.47044	4.26387	0.5190
4	3.145	VV	0.1424	23.84449	2.23623	0.2721
5	3.360	VB	0.1439	49.40802	4.57715	0.5639
6	3.835	BB	0.1245	15.77759	1.79521	0.1801
7	4.726	BB	0.1003	84.84641	12.33343	0.9684
8	5.708	BB	0.1068	14.26201	2.05921	0.1628
Totals :				8761.54551	2049.16687	

Chromatogram and HPLC integrations of **H2j**.

5. References

1. Chin, E.-Z.; Tan, S.-P.; Liew, S.-Y.; Kurz, T. *Malaysian J. Chem.* **2021,** *23,* 19–25. doi:10.1055/a-1330-4198
2. Wang, M. X.; Lin, S. J. *J. Org. Chem.* **2002,** *67,* 6542–6545. doi:10.1021/jo0256282
3. Harada, K. *Nature* **1963,** *4912,* 1201. doi:10.1038/2001201a0
